# Aero-YOLO26: A Lightweight Detector for Small-Object Detection in UAV-Acquired Images with Collaborative Feature Optimization

**DOI:** 10.3390/s26196346

**Published:** 2026-10-08

**Authors:** Zixiang Feng, Jiaying Li, Jialei Xu, Rui Quan

**Affiliations:** Hubei Provincial Key Laboratory of High-Efficiency Solar Energy Utilization and Energy Storage Operation Control, Hubei University of Technology, Wuhan 430068, China; 2310221103@hbut.edu.cn (Z.F.); 2310231327@hbut.edu.cn (J.L.); 2510201317@hbut.edu.cn (J.X.)

**Keywords:** UAV-acquired imagery, Aero-YOLO26, lightweight neural network, aerial photography object detection

## Abstract

**Highlights:**

**What are the main findings?**

- Aero-YOLO26 is proposed with only 1.99 M parameters for UAV aerial small-target detection.
- Custom modules realize synergistic optimization of feature extraction, pooling, and fusion.

**What are the implications of the main findings?**

- It provides a novel paradigm for balancing detection accuracy and parameter compression in complex UAV aerial scenes.
- It provides a feasible technical basis for edge deployment on UAV platforms.

**Abstract:**

Low-altitude remote sensing utilizing unmanned aerial vehicles (UAVs) has evolved into a core pillar of the space–air–ground integrated perception architecture. However, research on detection tasks in UAV aerial imagery grapples with three principal hurdles: feature annihilation of small targets, insufficient robustness under intricate environmental variations, and constrained computational capacity of onboard edge devices. To tackle these issues, a lightweight aerial small-target detection model denoted as Aero-YOLO26 was proposed for aerial image object detection. It introduced a C2f_GhostECA lightweight feature extraction module and a SlimSPPF streamlined multi-scale pooling module, and optimized the global receptive field of the Efficient Channel Attention (ECA) mechanism. Furthermore, it reconstructed the backbone and Path Aggregation Network (PAN)–Feature Pyramid Network (FPN) fusion network, enabling lightweight co-optimization of extraction, pooling, and fusion. Experiments on the VisDrone2019 dataset show that Aero-YOLO26n reduces the parameter count by 22.6% compared with YOLO26n, while improving precision, recall, mAP@50, mAP@50–95, and F1 score by 14.9%, 15.2%, 11.4%, 11.7%, and 14.8%, respectively. On the self-built UAV-MCT dataset, it attains 85.61% precision, 83.78% recall, mAP@50 of 90.82%, and mAP@50-95 of 59.60%, with average precision (AP) for cars and pedestrians reaching 99.4% and 96.6%. The proposed lightweight computer-vision object detection model using UAV-acquired imagery markedly bolsters the detection performance for small objects in UAV-acquired aerial imagery while achieving extreme model size compression, and its excellent performance demonstrates its potential for a wide range of application scenarios.

## 1. Introduction

With the rapid advancement of urbanization and smart city construction, the space–air–ground integrated perception system has become the core support for refined urban governance. Within the broader field of unmanned aerial vehicle (UAV) remote sensing technology, leveraging the strengths of flexible deployment, high spatiotemporal resolution, and cost-effectiveness has gradually become a vital technical means for smart city management, traffic situation perception, and urban emergency response [1]. Traditional security monitoring systems suffer from inherent limitations, such as limited field of view and blind spots of fixed cameras, while UAV-based security monitoring systems can effectively solve these problems through aerial cruise and real-time monitoring. The theory of social remote sensing geo-computation proposed by Academician Li Deren et al. [2] states that remote sensing technology has developed from traditional Earth observation to the all-round observation of the Earth, people, and society. As a key node of the space–air–ground perception network, low-altitude UAV-acquired images can effectively compensate for the coverage blind spots of ground-based fixed monitoring equipment and realize dynamic and refined perception of the entire urban area. Concurrently, the accelerated evolution of autonomous driving technology has raised higher requirements for the real-time environmental perception capability of vehicles. However, traditional vehicle-mounted perception systems are constrained by view occlusion and limited detection range, making it difficult to cope with blind spot problems in complex urban scenarios. With the advantages of flexibility, wide coverage, and high maneuverability, UAVs can not only provide dynamic monitoring means for smart city management, but also offer a God’s-eye view airborne perception supplement for autonomous driving vehicles [3].

Recently, small-object detection in UAV-acquired images has been challenged by three fundamental difficulties, including feature annihilation of small targets, insufficient robustness in complex scenarios, and constraints of edge-deployment computing power. The pixel proportion of targets captured by UAVs at high altitude is extremely low (smaller than 32 × 32 pixels), accompanied by weak target signals, limited information content, and strong background interference, which jointly increase the difficulty of small-target detection [4]. In addition, the sequential five downsampling operations, corresponding to a cumulative stride of 32 in the native YOLO backbone, cause the complete disappearance of fine spatial cues for small objects at deeper feature levels. Additionally, UAV perspectives have problems such as arbitrary target rotation, uneven illumination, cluttered backgrounds, and target occlusion. Conventional convolution and fixed interpolation upsampling struggle to characterize deformed and blurred targets, leading to frequent misclassifications and missed detections [5]. Furthermore, UAV onboard equipment belongs to the edge end, with limited onboard computing power, which requires the model to be lightweight and efficient, strictly control model size and computational budget (GFLOPs), and reconcile accuracy with efficiency [6].

To address the issue of small-target feature annihilation, existing research mainly alleviated fine-detail loss from extensive downsampling through retaining high-resolution features, optimizing multi-scale fusion, or incorporating an additional small-object detection head. Cheng et al. [7] systematically reviewed the technical context of bulk small-object detection, explicitly pinpointed detail erosion from downsampling as the central impediment, and summarized the improvement strategies of existing methods from six dimensions, including image quality degradation, scale and viewing angle changes, and small-target detection difficulties. Wang et al. [8] chronicled the evolution of overhead small-object detection using deep neural networks, classified and elaborated various methods from the aspects of learning discriminative features, applying super-resolution techniques, and achieving real-time lightweight detection, and pointed out that high-resolution feature retention is the key optimization direction. Tian et al. [9] proposed the YOLOv12 framework with an attention mechanism as the core, whose primary innovation is to build an attention-centric real-time detection architecture, fully harnessing its attention mechanism’s strengths while keeping inference speeds near CNN-based models. Shakya et al. [10] conducted a systematic benchmark test and Pareto efficiency comparison of streamlined YOLO variants on UAV and remote sensing datasets, providing a reference for model selection for small-target detection on edge devices. Gong et al. [11] proposed the AED-YOLO11 model with YOLO11 as the baseline, realized dynamic aggregation of frequency-domain features by designing an AFDA module, and optimized the detection head structure to better match small-object needs, verifying the improvement effect of the frequency-domain enhancement strategy on the recall rate of small objects. Bi et al. [12] designed SPDC-YOLO, which discarded the last C2f component and the ultimate downsampling step from the YOLOv8 spine, replaced them with the CGB downsampling module, and introduced the PPA parallel block perception attention module to intensify small-object features, effectively alleviating the attenuation of spatial details in the deep network. Li et al. [13] presented SOD-YOLO, which embedded the RFCBAM within the spine, and designed the BSSI-FPN, which improved the performance of YOLOv8 in detection of small targets for UAV aerial imagery. Xie et al. [14] proposed a density-guided two-stage detection framework, DG-TSOD, designed a dense target region searcher to focus on high-density areas, and improved the recognition effect of aerial dense small targets through coarse-fine granularity collaborative detection. Wei et al. [15] proposed DV-DETR with RT-DETR as the baseline, which enhanced the feature modeling ability of small-size targets in high-density scenarios by introducing a recalibration attention unit and deformable attention mechanism. Wang et al. [16] introduced UAV-YOLOv8, incorporating the BiFormer attention mechanism to refine the backbone, combined with a Wise-IoU box regression loss, which was exclusively suited for challenging tiny-object detection in drone-captured scenes. These investigations have ameliorated the feature representation of challenging small objects from different technical paths, but some schemes still mainly rely on adding detection heads or upsampling for remediation, and fail to systematically solve the problem of spatial detail attenuation in deep networks at the source of feature encoding.

Aiming at the problem of insufficient robustness in complex scenarios, current research mainly focuses on rotating target adaptation, attention mechanism enhancement, background noise suppression, or loss function optimization to boost the model’s anti-interference capability. Liu et al. [17] advocated RT-DETR-based WDFS-DETR by integrating wavelet transform convolution and a two-stage adaptive normalization mechanism, which effectively enhanced the feature modeling ability of multi-scale attention for aerial small targets. Zhang et al. [18] proposed an ASAHI method aiming to reduce redundant computation in high-resolution remote sensing images, which greatly reduced the redundant computation of inference through the adaptive slice size strategy. Sen et al. [19] incorporated the SimAM global attention into the backbone and designed an ELAN-DW module and an improved KFDIOU loss function based on the YOLO11-OBB framework, which effectively adapted to the detection of multi-directional rotating targets in power inspection scenarios. Zhu et al. [20] proposed a UAV rotating target detection system founded on key-point representation, simplifying the regression process of the rotating box through key-point prediction and rotating distance IoU loss, reducing the computational cost. Wang et al. [21] proposed ECA-Net to realize local cross-channel interaction through one-dimensional convolution, and brought significant performance gain with only a small number of parameters (k < 9), providing a lightweight scheme for the efficient deployment of channel attention in resource-constrained scenarios. Zhao et al. [22] proposed a UFDT-YOLO detection framework for harsh foggy environments, crafting a multi-branch learning framework from YOLOv12, and integrating the joint optimization strategy of end-to-end image enhancement restoration and target detection. Qiu et al. [23] proposed YOLO-Air by designing an ASFM adaptive scale fusion module and suppressing background noise from both spatial and semantic dimensions through effective multi-scale feature fusion and an adaptive channel attention mechanism. Zhang et al. [24] developed the MISATrack model by integrating a Frequency-Domain Encoder (FDE) and a Scale-Frequency Linear Attention (SFLA) mechanism, and achieved a balance between effectiveness and efficiency on multiple UAV tracking benchmarks through multi-band visual information fusion and efficient hierarchical feature interaction. Ma et al. [25] advanced DDF-YOLO, productively capturing anomalies in irregularly shaped forms by designing the C2f-DCNv4 dynamic deformable convolution module, introduced the DySample dynamic upsampling strategy to optimize multi-scale feature fusion to retain key shallow spatial details, and adopted the adaptive Focaler-ECIoU loss function to dynamically adjust the sample weight during the bounding box regression process. Shao et al. [26] crafted Aero-YOLO based on the YOLOv8 framework, superseded the original Conv module with GSConv and the C2f module with C3, and introduced a hybrid attention mechanism of CoordAtt and ShuffleAttention. In all, such studies have effectively improved the adaptability of the model to rotation, occlusion, and cluttered backgrounds, but most schemes still rely on fixed receptive field design and static attention weight allocation mechanisms, and there remains scope for further enhancement when applied to real-world scenarios with drastic changes in target scale and dynamic, complex backgrounds in aerial images.

Aiming at the problem of limited onboard edge computing power, existing research focused on lightweight transformation and obtained an optimal trade-off between precision and computational complexity by simplifying modules, replacing convolution, and scaling the network. Murat et al. [27] systematically reviewed the technical evolution and performance comparison of each version from YOLOv1 to YOLOv11, and UAV application scenarios took lightweight design and small-object detection as their most essential development requirements. Wan et al. [28] formulated the DAU-YOLO algorithm on the basis of YOLOv8s, employing lightweight modules to reconstruct the small-target detection network for efficient adaptation to the onboard low-computing-power platform. Han et al. [29] proposed GhostNet, which generated redundant feature maps through native convolution and cheap linear transformation, dramatically curbing the computational expense while preserving representational capability, and providing a classic paradigm for lightweight network design. Zhang [30] proposed Drone-YOLO, which integrated the GhostNet lightweight strategy and a hybrid attention mechanism, and achieved efficient UAV target detection with a small number of parameters. Lei et al. [31] suggested RPS-YOLO to replace the classic feature hierarchy with an RFP structure, realizing lightweight detection of UAV small targets on the YOLOv8 architecture, and effectively coordinating reliability with computational efficiency on resource-constrained edge devices. Zhang et al. [32] proposed SWL-YOLO, which integrated SAFM, an LSK mechanism, and wavelet downsampling based on YOLOv11, achieving lightweight and high-precision detection of remote sensing small targets. Xu et al. [33] proposed a YOLO-HyperVision method, which used a Vision Transformer as the backbone to enhance the global modeling ability of YOLOv5 for dynamic traffic information detection scenarios. Yue et al. [34] devised LE-YOLO by taking the YOLOv8n architecture as the foundation and designed the LHGNet streamlined backbone and optimized the feature map size and structure, which is specially customized for tiny-target detection in UAV edge deployment. Chen et al. [35] proposed a DTSSNet model, which optimized the training efficiency of UAV target detection from both sample and feature dimensions through a dynamic-training sample-selection strategy and an attention-enhanced feature module. Min et al. [36] developed the LWUAVDet compact framework, designed an E-FPN feature fusion structure and a PixED detection head, which enabled instant UAV detection on edge hardware and balanced processing limits with identification fidelity. Bakirci [37] built an autonomous UAV platform vehicle detection system based on YOLOv8, proving the practical efficacy of the compressed model within intelligent transportation frameworks. Although the above lightweight designs reduce computational burden for potential edge deployment, achieving reliable onboard inference within complex dynamic scenes still calls for advances in spatiotemporal perception. This motivates research integrating spatio-temporal intelligence into the edge-computing pipelines. Vankdoth et al. [38] proposed a deep intelligent transport solution for travel duration prediction leveraging spatio-temporal data, crafting a hybrid deep scheme for precise estimation, providing a spatio-temporal data modeling paradigm for UAV target detection and edge deployment in intelligent transportation scenarios. Li et al. [39] pioneered the cutting-edge research perspective of spatio-temporal intelligence (STI), systematically constructed a unified paradigm framework of perceptual intelligence, cognitive intelligence, and behavioral intelligence, and clarified the technical path of spatio-temporal feature modeling and global perception, laying a theoretical foundation for UAV multi-scale and time-series aerial perception. Dong et al. [40] proposed a novel next-generation paradigm toward spatiotemporal intelligence in neural city, which unified multi-source urban heterogeneous data with a differentiable spatio-temporal feature field, and integrated UAV aerial survey technology to realize refined global monitoring of urban space. Liang et al. [41] conceived a streamlined model drawing on improved YOLO11 for the target detection task for vehicle–road collaborative scenarios, and addressed challenges including unsatisfactory detection precision, false alarms and target omissions, and lightweight scenario modeling in autonomous driving. Although the above lightweight schemes were continuously iterated, most of them only relied on single-structure replacement and failed to realize the lightweight collaborative optimization of the entire pipeline of feature extraction, pooling, and fusion.

Therefore, designing a UAV-acquired image small-object detection model that is robust to viewpoint and appearance variations within the horizontal bounding-box detection setting, has strong small-target detection capability, and can resist complex background interference has important theoretical value and practical significance.

Specifically, the YOLO algorithmic lineage has recently undergone ongoing evolution. YOLOv8 [42] improved detection accuracy through the C2f module and decoupled head, but there is still considerable room for optimization. YOLOv11 [43] introduced the C3k2 module and C2PSA attention mechanism. In January 2026, Ultralytics released YOLO26 [44] (also known as YOLOv26), the latest YOLO iteration, designed specifically for edge and low-power devices. The core innovation of YOLO26 is that it removes the distribution focal loss (DFL) module to simplify export, enables native non-maximum suppression (NMS)-free end-to-end inference to reduce latency, applies progressive loss (ProgLoss) and small-target-aware label assignment (STAL) to enhance small-target recognition, and uses the MuSGD optimizer to accelerate convergence.

With regard to the aforementioned issues, this study puts forward the Aero-YOLO26 model for aerial photography small-target detection. Based on the YOLO26 architecture, the model introduces three lightweight optimizations and verifies its effectiveness through the recognized benchmark dataset VisDrone2019 [45] and the self-built UAV-MCT dataset. Finally, its potential application in two typical scenarios is discussed, including smart city management inspection and autonomous driving airborne perception. It should be clarified that the proposed Aero-YOLO26 is optimized for visual characteristics of UAV-captured RGB aerial images, such as densely distributed small-scale targets, drastic object-scale variation, and cluttered backgrounds. This detector does not take flight altitude, ground sample distance (GSD), GPS/IMU telemetry, or other UAV-platform-specific sensor data as network inputs. All performance gains originate from pixel-level visual-feature optimization rather than auxiliary platform telemetry information. The principal contributions of this study are summarized below:

(1) A hierarchical C2f_GhostECA lightweight feature extraction and fusion module is proposed. Enhanced ECA is embedded into GhostBottleneck and further incorporated into the C2f structure, forming a hierarchical optimization from channel attention and lightweight bottleneck extraction to multi-branch feature fusion. This design reduces redundant feature computation while enhancing small-target representations and suppressing complex background interference.
(2) A SlimSPPF multi-scale pooling module is developed, which compresses feature channels before multi-scale pooling, thereby reducing the memory-access cost of pooling branches while preserving the multi-scale receptive-field coverage of the original SPPF. Additionally, it improves contextual modeling for targets with significant scale variations and enhances the model’s suitability for prospective UAV edge-deployment scenarios.
(3) The backbone and PAN-FPN feature fusion network are reconstructed, with recalibrated depth and width scaling coefficients to establish an optimal equilibrium between accuracy and model efficiency.
(4) Comprehensive evaluations are conducted on the public VisDrone2019 benchmark and the self-built UAV-MCT dataset. Experimental results verify that the proposed Aero-YOLO26 detector, a lightweight computer-vision object detection model using UAV-acquired imagery, achieves prominent small-object detection performance under extreme model-size compression, demonstrating considerable application potential for various practical UAV airborne perception tasks in smart-city scenarios.

## 2. Materials and Methods

### 2.1. Neural Network Architecture of Aero-YOLO26

**Figure 1:**
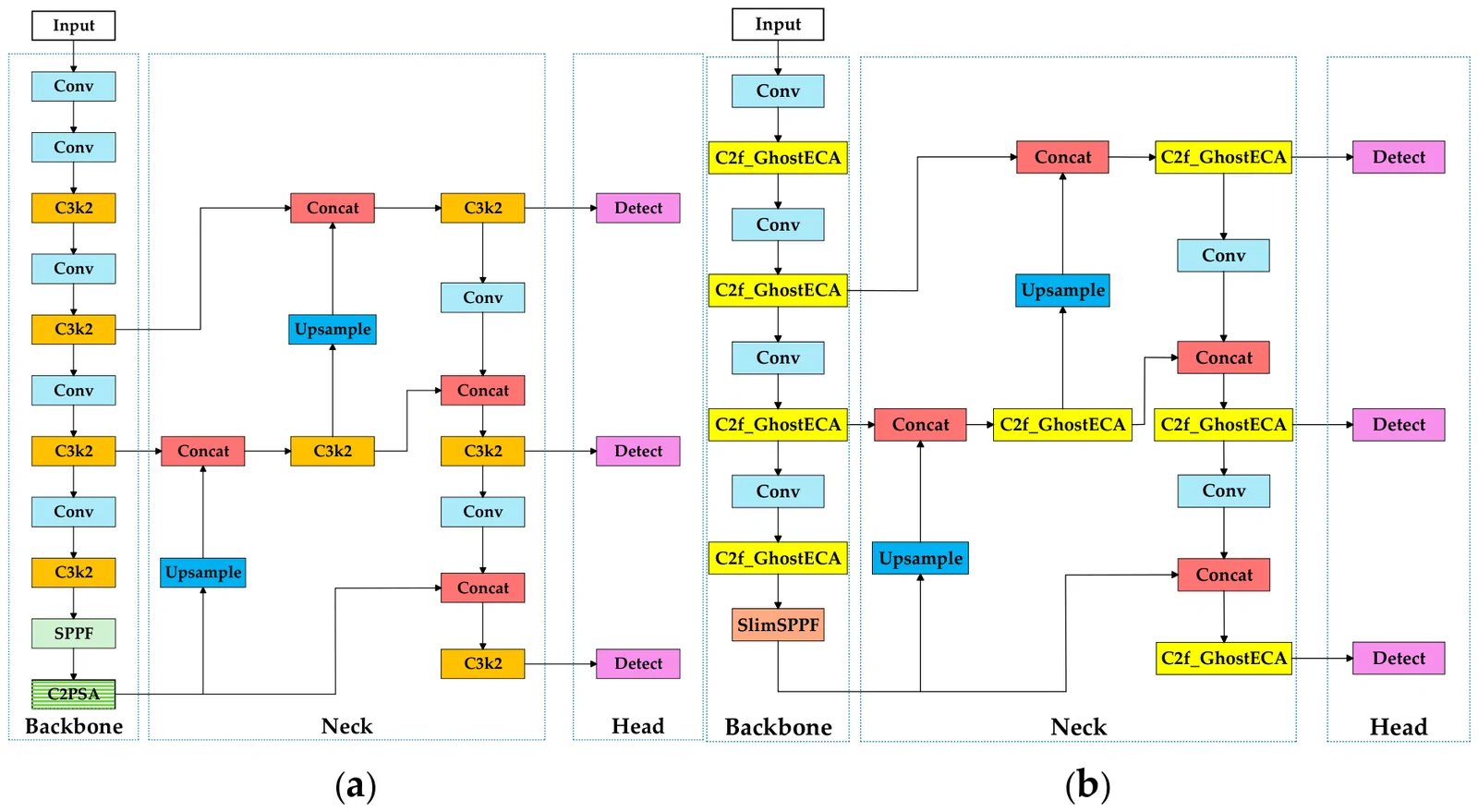
Neural network structure diagram: (**a**) YOLO26′s principal structure diagram; (**b**) Aero-YOLO26′s principal structure diagram.

Aero-YOLO26 is an improved version of YOLO26 optimized for detection tasks in UAV aerial captures, with its architecture illustrated in Figure 1. Aiming at the three core challenges of dense small targets, large scale differences, and limited end-side computing power in aerial photography scenarios, the model fully reconstructs the core feature extraction and fusion modules on the basis of retaining the compatibility of YOLO26 native detection head and training framework. Through the collaborative optimization of lightweight convolution, enhanced attention, and streamlined multi-scale pooling, it substantially enhances object detection performance while improving edge-deployment speed, especially adapting to the detection of long-distance, densely distributed targets such as vehicles and pedestrians from the UAV perspective. The concrete realization and efficacy of these optimizations are detailed in the following subsections.

The overall framework of Aero-YOLO26 is partitioned into four functional units: input, backbone network, neck network, and detection head. Regarding the aforementioned structural components, the input module is dedicated to implementing image resizing, data enhancement, adaptive anchor box derivation, and adaptive spatial adjustment, with 640 × 640 × 3 defined as the base input resolution. The backbone network is constructed with convolutional layers, C2f_GhostECA modules, and SlimSPPF modules in series to complete shallow texture extraction, middle-layer feature enhancement, and terminal multi-scale feature aggregation. For aligning and enriching multi-scale features, the neck architecture integrates a circular fusion structure with upsampling, feature concatenation, and C2f_GhostECA modules. The detection head retains the native anchor-free decoupled Detect head of YOLO26 to ensure the compatibility of inference logic and framework. Compared to generic models including YOLO26, the proposed Aero-YOLO26 achieves prominent improvement in identifying aerial small targets, and it meanwhile reduces the parameter count by approximately 22.6%, thus reconciling detection performance with deployment cost in an optimal manner.

As the newest iteration of single-stage object detection models, YOLO26 was first unveiled by the Ultralytics team in 2026 and has reached an unprecedented state-of-the-art in runtime efficiency and precision. Combined with the performance requirements and resource constraints of UAV aerial target detection, this study selects YOLO26 as the base model. As a cutting-edge end-to-end detection model, YOLO26 provides Nano (n), Small (s), Medium (m), Large (l), and Extra Large (x) variants to adapt to different computing power scenarios, and has made a number of key optimizations in structure. YOLO26 applies the C3k2 fundamental block to substitute the older C2f for more efficient feature extraction; implements an anchor-free decoupled head design to isolate classification from regression tasks, and introduces an E2E end-to-end loss function to simplify the post-processing pipeline. In addition, it optimizes the data augmentation strategy and learning rate scheduling mechanism to further boost general applicability, and adopts NMS post-processing when outputting bounding boxes to filter invalid detection results, which reduces redundant overlapping boxes and improves detection accuracy.

Despite the excellent performance of YOLO26, its design is oriented to general detection scenarios and has obvious deficiencies for aerial photography scenarios. First, the standard convolution and C3k2 modules possess substantial parameter volumes, obstructing direct installation on nimble, lightweight UAVs. Second, the conventional attention mechanism has a limited receptive field, and it suffers from insufficient capability in capturing global features for remote tiny objects. Finally, the native SPPF module has low multi-scale fusion efficiency, which makes it difficult to cover the large-scale variations in aerial photography scenarios. To solve the above problems, Aero-YOLO26 carries out directional improvements on the core modules of YOLO26 from the perspective of lightweight network reconstruction, feature extraction enhancement, and multi-scale fusion optimization.

### 2.2. Enhanced ECA Channel Attention Module

**Figure 2:**
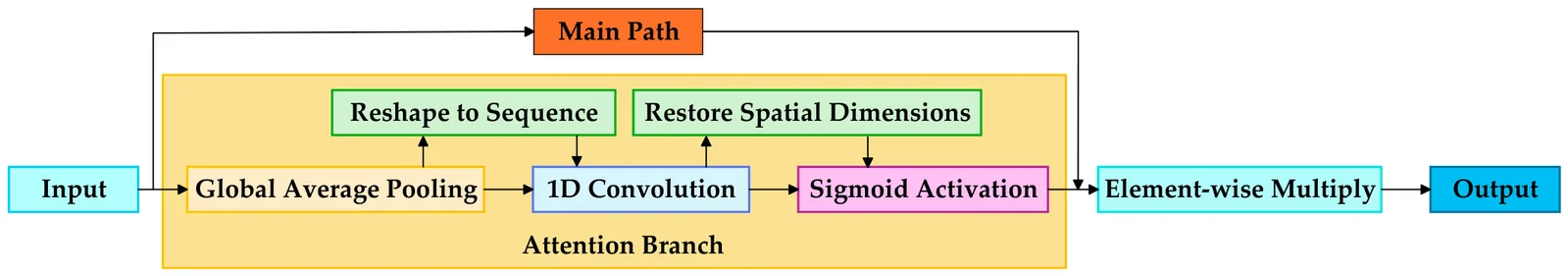
Structural block diagram of Enhanced ECA module.

The standard Efficient Channel Attention (ECA) module [21], with its default local cross-channel interaction kernel size of *k* = 3, is optimized for general-purpose visual recognition tasks where objects occupy substantial image regions. For UAV aerial targets, which typically occupy only a few pixels and are deeply entangled with background clutter, this limited receptive field proves inadequate for capturing the long-range channel dependencies critical for distinguishing weak signals from noise. Simply enlarging the convolution kernel would introduce additional parameters and computational overhead, contradicting the lightweight deployment requirement. To address this dilemma without sacrificing efficiency, an Enhanced ECA (EECA) module is designed, which expands the convolution kernel size from 3 to 7, broadening the channel-wise receptive field and enabling longer-range channel interactions. As presented in Figure 2, the detailed architecture comprises global adaptive average pooling, 1D convolution with kernel size 7, and sigmoid activation. Notably, no channel dimension reduction is performed throughout this process, ensuring that the overall parameter overhead remains extremely low. Consequently, the EECA module strengthens the response of small-target-relevant channels while suppressing noise-dominated ones, achieving improved feature discriminability with negligible extra parameter cost.

Let the input feature map *X* ∈ ℝ*^C^*^×^*^H^*^×^*^W^*, with C as the channel number, and *H* and *W* as spatial dimensions. The channel attention vector and the recalibrated output are computed as

(1) $$\mathrm{EEC}A_{k}(X)=\sigma \left(\mathrm{Conv}1D_{k=7}{\left(G(X{)}^{⊤}\right)}^{⊤}\right)⊙X$$

where 𝒢(*X*) denotes global average pooling, 𝒢(*X*) ∈ ℝ*^C^*. *Conv*1*D_k_*(⋅) represents one-dimensional convolution with kernel size *k*, acting on a channel-dimension-oriented sequence. The transpose operation (⋅)^⊤^ is used to adjust the dimension to match the input and output format of the convolution.

The EECA module enhances small-target feature discriminability through lightweight channel-wise receptive field expansion. Nevertheless, attention recalibration alone cannot eliminate the inherent parameter redundancy of the standard bottlenecks in YOLO26. To address both attention enhancement and parameter compression simultaneously within the bottleneck structure, the EECA module is integrated with Ghost convolution to construct the GhostBottleneck_ECA module, as detailed in the following subsection.

### 2.3. GhostBottleneck_ECA Lightweight Attention Bottleneck Module

**Figure 3:**
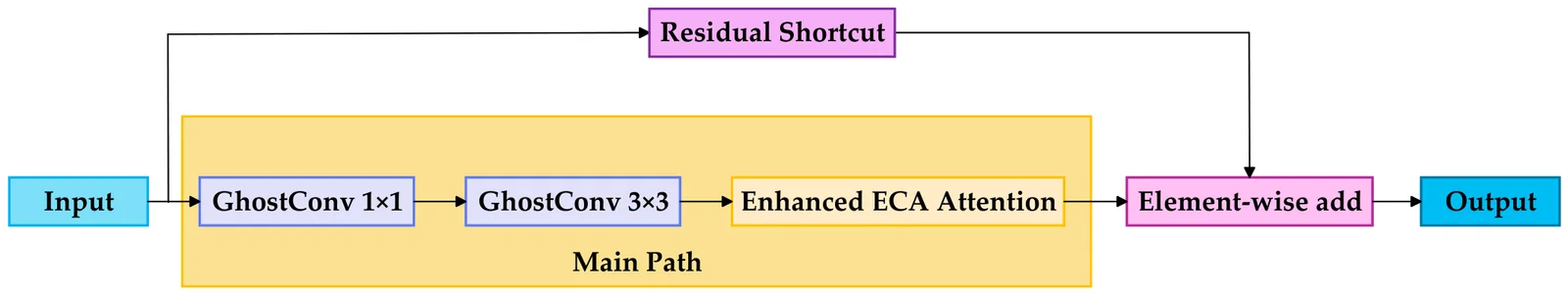
Structural block diagram of GhostBottleneck_ECA lightweight attention bottleneck module.

In the original backbone and neck of YOLO26, abundant redundancy exists within the intermediate feature maps generated by standard convolutions and C3k2 modules, leading to unnecessary computational overhead. Ghost convolution [29] serves as a prevalent lightweight network optimization paradigm, which reduces parameter scale by generating redundant feature maps through low-cost linear transformations based on a limited number of intrinsic features. This design implicitly assumes that both intrinsic and synthetic redundant features contribute equally to downstream detection tasks. However, for UAV small-target detection scenarios, the inexpensive depthwise convolution transformations indiscriminately propagate task-relevant signals and background noise, further degrading the inherently low signal-to-noise ratio of tiny aerial targets. The detailed architecture is visualized in Figure 3; input feature embeddings enter the main path and sequentially pass through a 1 × 1 Ghost convolution layer and a 3 × 3 Ghost convolution layer for lightweight feature extraction. The extracted features are then fed into the EECA module to conduct adaptive channel screening, suppressing background noise and amplifying target-related feature responses. Meanwhile, the original input feature map is delivered via a residual shortcut branch. Finally, features from the main path and shortcut branch are fused via element-wise addition to generate the output feature. This architecture delivers three complementary advantages. First, extreme lightweight properties are realized via Ghost convolution, which is intended to reduce the parameter count relative to standard convolution. Second, the embedded EECA module filters complex background noise and enhances the representation capability of weak small-target features in aerial scenes. Third, the residual shortcut connection alleviates the inherent gradient vanishing problem of deep neural networks and improves the overall stability of feature extraction. In all, these combined benefits establish a favorable trade-off between parameter efficiency and feature representation capability, effectively alleviating the feature annihilation problem in UAV aerial object perception tasks.

For GhostConv, let the input $X\in R^{C_{\mathrm{in}}\times H\times W}$, if input channels match output channels and shortcut = True, the final output is calculated by

*Y* = *X* + *EEC*A(GhostConv_3×3_(GhostConv_1×1_(*X*))) (2)

If shortcut = False or the number of channels does not match, then

*Y* = *EEC*A(GhostConv_3×3_(GhostConv_1×1_(*X*))) (3)

GhostBottleneck_ECA replaces the standard bottleneck with two GhostConv layers followed by EECA channel recalibration, and a residual connection is retained when input and output dimensions match. This module is used as the internal bottleneck of C2f_GhostECA to jointly reduce parameters and recalibrate channel responses.

The GhostBottleneck_ECA module optimizes lightweighting and attention enhancement at the level of a single bottleneck unit. However, in the YOLO26 architecture, the C2f module acts as the core component for multi-scale feature fusion in the neck network, which aggregates features by cascading multiple bottlenecks through a split–merge paradigm. To extend the lightweight and anti-interference advantages of GhostBottleneck_ECA from a single computational unit to the entire feature fusion pipeline, a modified C2f_GhostECA module is constructed by replacing all internal standard bottlenecks with GhostBottleneck_ECA modules.

### 2.4. C2f_GhostECA Core Feature Fusion Module

**Figure 4:**
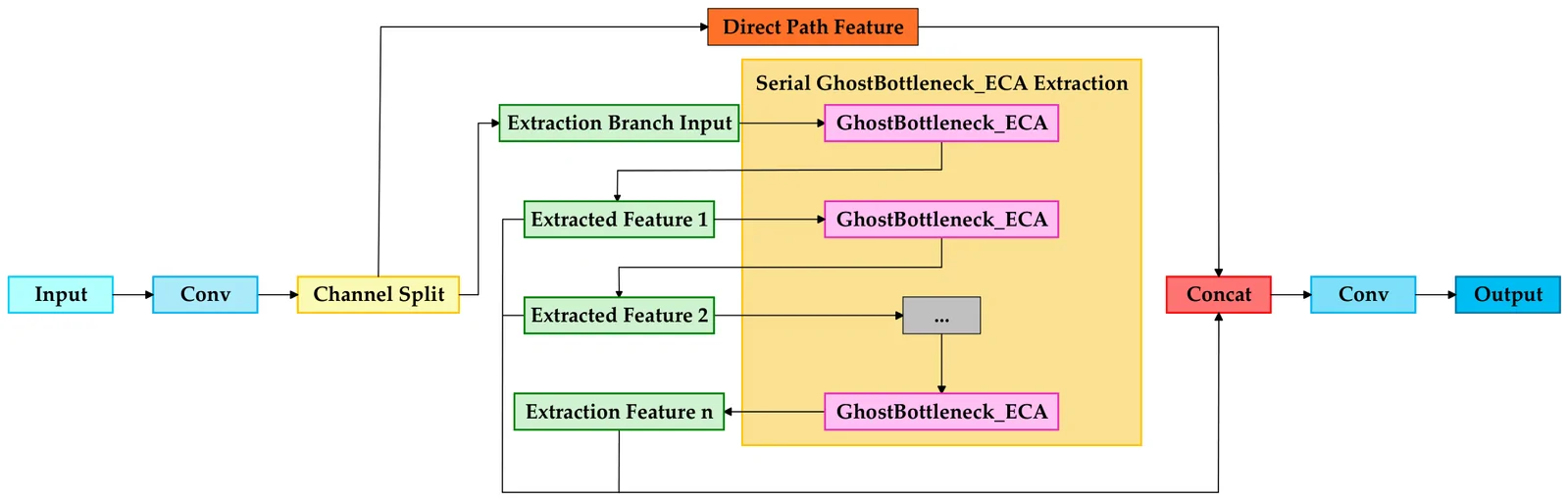
Structural block diagram of C2f_GhostECA core feature fusion module.

As a classic split–process–merge architecture within the YOLO family, the C2f module serves as the fundamental building block for multi-scale feature fusion within the neck network, providing efficient feature aggregation. The native C2f module adopted by YOLO26 relies on standard Bottleneck blocks, which introduce excessive parameter overhead and impede lightweight edge-side deployment. Meanwhile, a naive lightweighting scheme that simply replaces standard Bottlenecks with Ghost convolutions inevitably degrades feature discriminability, which severely impairs detection performance for scale-varying small aerial targets. To tackle these challenges, the native C2f module of YOLO26 is reconstructed by substituting all internal standard Bottlenecks with the proposed GhostBottleneck_ECA modules, constructing the improved C2f_GhostECA module, which achieves comprehensive lightweight optimization and attention-driven feature enhancement. The detailed architecture of C2f_GhostECA is visualized in Figure 4. Specifically, the input feature map is first split via a 1 × 1 convolution layer, and multi-branch feature extraction is subsequently performed by stacked GhostBottleneck_ECA modules; the processed multi-scale features are finally concatenated for subsequent detection.

Let the input $X\in R^{C_{\mathrm{in}}\times H\times W}$, the resulting features are concatenated and projected to the output dimension:

(4) $$Y={\mathrm{Conv}}_{1\times 1}^{\left(b\right)}\left(\mathrm{Concat}(T_{1},B_{1},B_{2},\ldots ,B_{n})\right)$$

(5) $$\left[T_{1},T_{2}]=\mathrm{Split}({\mathrm{Conv}}_{1\times 1}^{\left(a\right)}(X)\right)$$

*B*_1_ = Gb(*T*_2_), *B_i_* = Gb (*B_i_*_−1_), *i* = 2, 3, …, *n* (6)

where *T*_1_ denotes the shortcut branch obtained from channel-dimension splitting, which skips the stacked GhostBottleneck_ECA processing, and *T*_2_ represents the processing branch that is fed into successive GhostBottleneck_ECA units. Split(⋅) denotes the tensor splitting operation performed along the channel dimension. Gb(⋅) represents the GhostBottleneck_ECA unit. *n* stands for the number of stacked GhostBottleneck_ECA blocks inside the C2f_GhostECA module, and *B_i_* indicates the output feature map produced by the *i*-th stacked GhostBottleneck_ECA. Concat(⋅) denotes channel-wise feature concatenation. ${\mathrm{Conv}}_{1\times 1}^{\left(a\right)}$ is the input 1 × 1 convolution for channel transformation prior to tensor splitting, and ${\mathrm{Conv}}_{1\times 1}^{\left(b\right)}$ is the output projection 1 × 1 convolution that maps the concatenated multi-branch features to the target output-channel dimension.

Benefiting from this structural redesign, the C2f_GhostECA module achieves three complementary optimization objectives. First, embedded Ghost convolutions drastically reduce the overall parameter count of the neck network and alleviate computational bottlenecks for UAV edge-side deployment. Second, the internal EECA modules perform channel recalibration within the feature maps at different pyramid levels, aligning features from different feature pyramid levels before they are fed into the detection head. Third, the inherent multi-branch split–process–merge paradigm of C2f cooperates with attention-enhanced bottlenecks to simultaneously capture fine-grained spatial details and global semantic context. Overall, the redesigned C2f_GhostECA module achieves a favorable balance between computational tractability and semantic feature coherence, systematically optimizing the multi-scale feature fusion pipeline tailored for UAV small-object detection.

The C2f_GhostECA module optimizes multi-branch feature fusion within the neck network through channel-wise recalibration and lightweight bottleneck replacement. However, at the terminal of the backbone, a distinct but complementary function is performed by the Spatial Pyramid Pooling—Fast (SPPF) module, which aggregates contextual information across spatial scales. To ensure that this pooling stage does not become a memory-access bottleneck on UAV edge processors, the SlimSPPF module is proposed in the following subsection.

### 2.5. SlimSPPF Streamlined Multi-Scale Pooling Fusion Module

**Figure 5:**
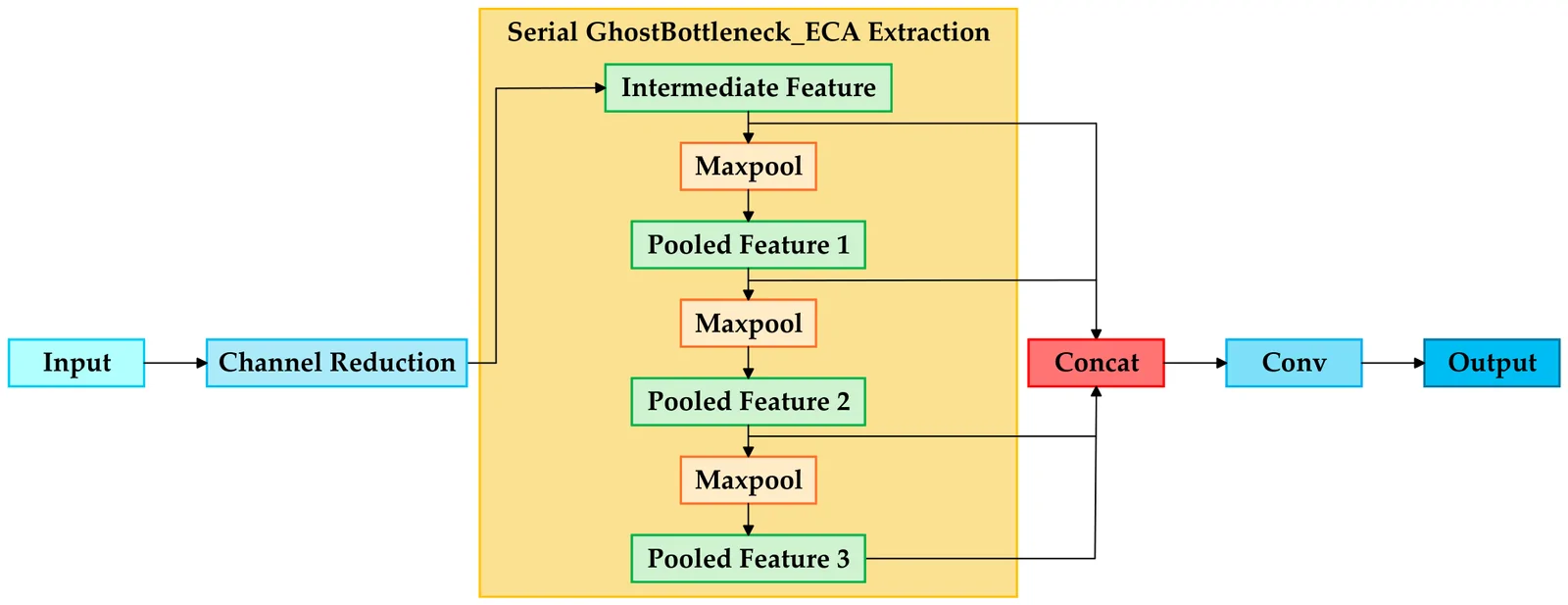
SlimSPPF streamlined multi-scale pooling fusion module structure diagram.

The standard Spatial Pyramid Pooling—Fast (SPPF) module aggregates multi-scale contextual information through cascaded max-pooling operations on the full channel dimension of input feature maps. Although this structure effectively expands the network receptive field, the native SPPF suffers from channel redundancy and low multi-scale fusion efficiency, making it poorly adapted to the drastic scale variations observed in UAV aerial imagery [46]. More importantly, pooling operations conducted on high-dimensional feature maps introduce substantial memory access overhead, which severely limits deployment on resource-constrained UAV edge processors. To tackle such hardware-level constraints, a SlimSPPF module is constructed to replace the original SPPF and serve as the multi-scale feature aggregation unit at the backbone terminal, and its detailed architecture is illustrated in Figure 5. SlimSPPF adopts a channel pre-compression strategy. A 1 × 1 convolution first halves the channel dimension of the input feature map, substantially cutting the memory occupancy of all subsequent pooling operations. The compressed intermediate feature propagates through three successive cascaded 5 × 5 max-pooling layers to generate multi-scale pooled features. The original intermediate feature and the three groups of pooled features are subsequently concatenated. After multi-scale feature fusion, another 1 × 1 convolution projects the concatenated features back to the original channel dimension to produce the final output feature map.

Let the input X ∈ ℝ*^C^*^×^*^H^*^×^*^W^*, the output Y ∈ ℝ*^C^*^×^*^H^*^×^*^W^*:

Channel reduction:

*X*_1_ = Conv_1×1_(*X*) ∈ ℝ*^C^*^/2×^*^H^*^×^*^W^* (7)

Cascaded max pooling (kernel size k = 5, stride 1, padding 2):

(8) $$\begin{array}{c} P_{1}={\mathrm{MaxPool}}_{5\times 5}(X_{1})\\ P_{2}={\mathrm{MaxPool}}_{5\times 5}(P_{1})\\ P_{3}={\mathrm{MaxPool}}_{5\times 5}(P_{2}) \end{array}$$

Concatenation:

*C*_concat_ = Concat(*X*_1_,*P*_1_,*P*_2_,*P*_3_) ∈ ℝ^2*C*×^*^H^*^×^*^W^* (9)

Channel recovery:

*Y* = Conv_1×1_(*C*_concat_) ∈ ℝ*^C^*^×^*^H^*^×^*^W^* (10)

The final expression:

(11) $$Y={\mathrm{Conv}}_{1\times 1}^{\left(e\right)}\left(\mathrm{Concat}\left(X_{1},P_{1},P_{2},P_{3}\right)\right)$$

where ${\mathrm{Conv}}_{1\times 1}^{\left(e\right)}$ denotes the 1 × 1 channel recovery convolution (the superscript (*e*) stands for expansion).

Even though channel compression reduces the feature dimensions involved in pooling, two dedicated designs retain feature representation capability. Cascaded 5 × 5 max pooling (stride 1, padding 2) maintains the complete spatial receptive field coverage consistent with standard SPPF, and the final 1 × 1 convolution restores the original channel dimension, ensuring full feature representational capacity for subsequent neck modules. By introducing the channel pre-compression strategy, SlimSPPF is expected to reduce the memory-access burden of the pooling branches by approximately 30% compared with native SPPF, alleviating hardware buffer pressure on UAV edge chips while maintaining a comparable convolutional computational cost. Despite minor extra tensor movement overhead brought by feature concatenation, the bandwidth saved from lower feature buffer occupancy fully offsets such consumption on embedded platforms. Benefiting from this hardware-aware optimization, SlimSPPF retains equivalent multi-scale receptive field coverage and achieves stronger hardware adaptability for aerial scenes with drastic target scale variations. Serving as the terminal module of the backbone, it provides high-quality global multi-scale contextual features for downstream detection modules and meets the requirements of real-time onboard UAV inference.

In summary, the proposed Aero-YOLO26 model consists of the hierarchical C2f_GhostECA feature extraction and fusion module and the SlimSPPF multi-scale pooling module, and they are further integrated through the reconstructed backbone and PAN-FPN neck. The EECA module provides the foundational attention mechanism for channel-wise feature recalibration, and the GhostBottleneck_ECA module applies this attention mechanism at the single-bottleneck level, achieving a symbiotic integration of lightweight feature generation and noise suppression. The C2f_GhostECA module extends this integration to the multi-branch fusion architecture of the neck network, ensuring that the feature extraction and fusion stages are both computationally efficient and semantically coherent. Finally, the SlimSPPF module addresses the hardware-level memory access bottleneck at the backbone terminus, complementing the feature extraction optimizations with a hardware-aware multi-scale pooling strategy. Together, these modules achieve a full-pipeline co-optimization encompassing feature extraction, bottleneck lightweighting, multi-scale fusion, and pooling aggregation, a holistic strategy that distinguishes the proposed Aero-YOLO26 from existing methods that focus on isolated component improvements.

## 3. Experiments and Results

### 3.1. Datasets

To validate the performance of the proposed model, this study utilizes the publicly available VisDrone2019 dataset and the custom-developed UAV-MCT dataset.

As a widely recognized standard benchmark in UAV-centric computer vision research, the VisDrone2019 dataset was collected and sorted by the AISKYEYE team of the Machine Learning and Data Mining Laboratory of Tianjin University. The dataset covers diverse scenarios and environmental conditions, and faces the core challenges of high target density, large-scale changes, harsh weather, and low illumination in UAV images. It contains 6474 training images, 548 validation images, and 1610 test images, covering 10 target categories: pedestrian, person, bicycle, car, van, truck, tricycle, awning tricycle, bus, and motor.

**Figure 6:**
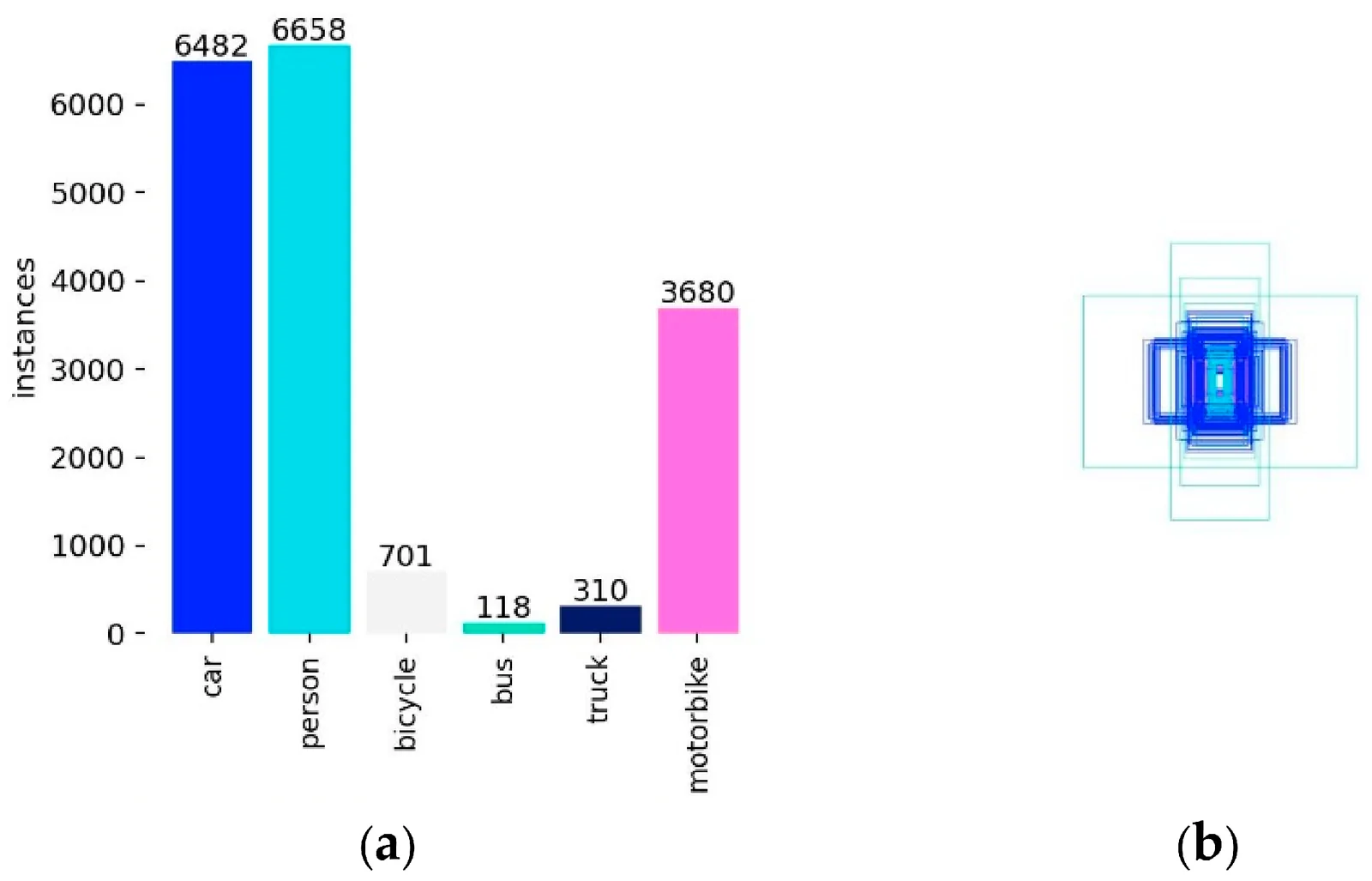
Category distribution and target box statistics of the UAV-MCT dataset: (**a**) class-wise instance count distribution; (**b**) bounding box spatial distribution.

In addition, a self-constructed UAV-MCT (UAV Multi-condition City Traffic) dataset is employed, which is captured via the DJI Mavic 3 Pro UAV in diverse real-world scenarios, and the category distribution of the UAV-MCT dataset is shown in Figure 6. In the UAV-MCT dataset, annotated objects are widely scattered across image frames without severe spatial clustering. Most ground-truth boxes correspond to small-size aerial targets, consistent with typical UAV overhead image characteristics.

The UAV-MCT dataset comprises 2355 high-resolution aerial images (5280 × 3956 pixels), acquired from altitudes ranging from approximately 30 to 60 m, covering a wide variety of environments, and including urban streets and university campuses. The shooting conditions are meticulously designed to encompass varying photometric factors, such as sufficient daylight, low-light nighttime conditions, cloudy weather, strong illumination, and significant glare, alongside various aerial perspectives. Moreover, all targets are annotated using the LabelImg tool. To ensure rigorous annotation consistency, a horizontal bounding box criterion is adopted, where only instances with more than 50% of their visible area are labeled, effectively handling occlusions. In total, 17,949 annotated instances are distributed across six categories, including car (6482), person (6658), motorbike (3680), bicycle (701), truck (310), and bus (118), as illustrated in Figure 6. To prevent data leakage in model evaluation, a sequence-based division strategy is applied according to temporal and scene continuity, which splits the dataset into 1600 images for training and 234 images for validation. In addition, this dataset also has an independent test set containing 521 images to objectively evaluate the final performance of the model. The dataset is scheduled to be made accessible to the public as open-source in the future upon request.

### 3.2. Experimental Environment

The experimental environment is built using PyTorch 2.6.0, accelerated by CUDA 12.4 on an NVIDIA GeForce RTX 4060 Laptop GPU. Model construction and training are completed based on the PyTorch framework, and the detailed configuration of the experimental environment is shown in Table 1.

### 3.3. Experimental Evaluation Metrics

The performance of the proposed method is evaluated in the experiment based on detection capability and computational complexity. Evaluation indices include precision (P), recall (R), average precision (AP), mean average precision (mAP), and F1 score, reflecting the model’s accuracy in predicting positive samples, the rate at which true positive samples are correctly identified, the model’s comprehensive detection performance for a single target category across all recall thresholds, the model’s overall detection accuracy across all target categories in multi-class detection tasks, and the model’s balanced detection performance by measuring the harmonic mean of precision and recall, respectively.

In multi-category target detection, predictions fall into four categories. An instance is classified as True Positive (TP) when predicted as positive while its ground truth is indeed positive; a case is categorized as False Positive (FP), commonly termed a false alarm, when a negative ground truth sample is incorrectly predicted as positive by the model; a result is identified as True Negative (TN) when both the predicted label and ground truth are negative; and an outcome is labeled as False Negative (FN), known as a missed detection, when a positive ground truth sample is incorrectly classified as negative. The calculation formulas of each metric are listed in Table 2 [47].

### 3.4. Experimental Results on VisDrone2019 Dataset

The evaluation encompasses two-stage architectures (Fast R-CNN, Mask R-CNN), mainstream YOLO-series models at nano, small, and medium scales (YOLOv8, YOLOv9, YOLOv11, and YOLO26), and recent lightweight detectors specifically designed for drone-captured imagery (YOLO-RACE [48], BAF-Net [49], Drone-YOLOn [30], Drone-YOLOt [30]). The proposed Aero-YOLO26 series, architected for extreme lightweight and accurate aerial photography scenarios, is introduced in two compact variants of Aero-YOLO26n and Aero-YOLO26s. The same training and evaluation settings are used for all compared models to ensure experimental reliability and fairness. The detailed training parameter settings are presented in Table 3. In addition, their accuracy and complexity metrics are reported in Table 4, focusing on precision, recall, mean average precision, number of parameters, and F1 score.

As shown in Table 4, Aero-YOLO26n contains 1.99 M parameters and achieves an mAP@50 of 0.382, whereas Aero-YOLO26s contains 9.16 M parameters and achieves an mAP@50 of 0.441. Among the evaluated small-scale models, Aero-YOLO26s obtains the highest precision, recall, mAP@50, mAP@50:95, and F1 score.

**Figure 7:**
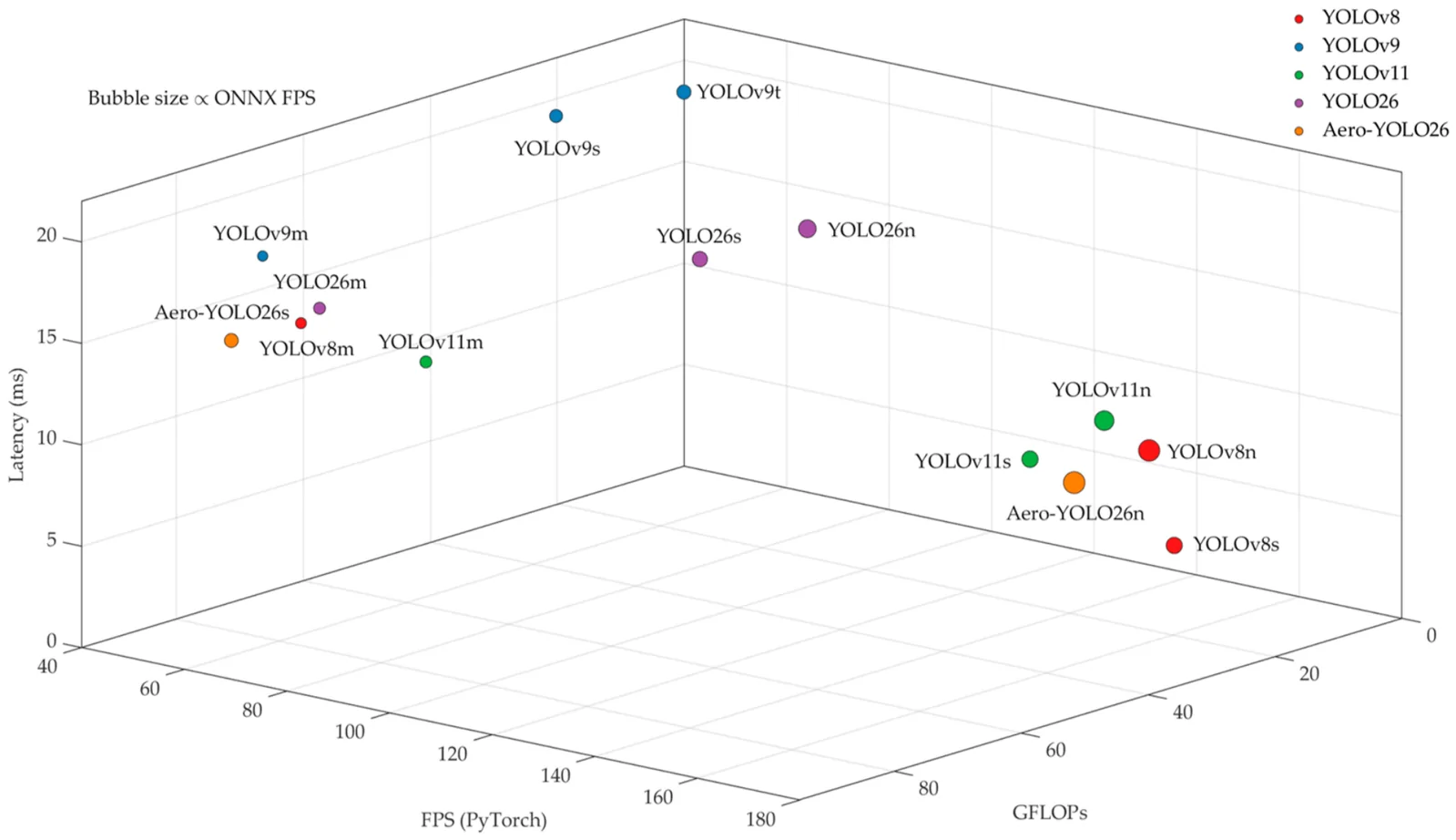
Trade-off among computational cost (GFLOPs), inference speed (FPS), latency, and ONNX throughput across YOLO-based detection models.

To complement the detection-accuracy comparison, the computational complexity and inference efficiency of the YOLO-based models are reported in Table 5 and visualized in Figure 7; bubble size encodes ONNX FPS, and color indicates model family.

The category-wise AP values of the nano-scale and small-scale models are listed in Table 6 and Table 7, respectively, showing whether the overall performance differences are consistent across object categories.

### 3.5. Experimental Results on UAV-MCT

The UAV-MCT experiment provides a complementary evaluation of Aero-YOLO26 under an independently collected UAV image distribution, and the purpose of this experiment is to examine whether the proposed model can maintain reliable detection performance in real-world application-oriented UAV scenes beyond the public benchmark.

**Figure 8:**
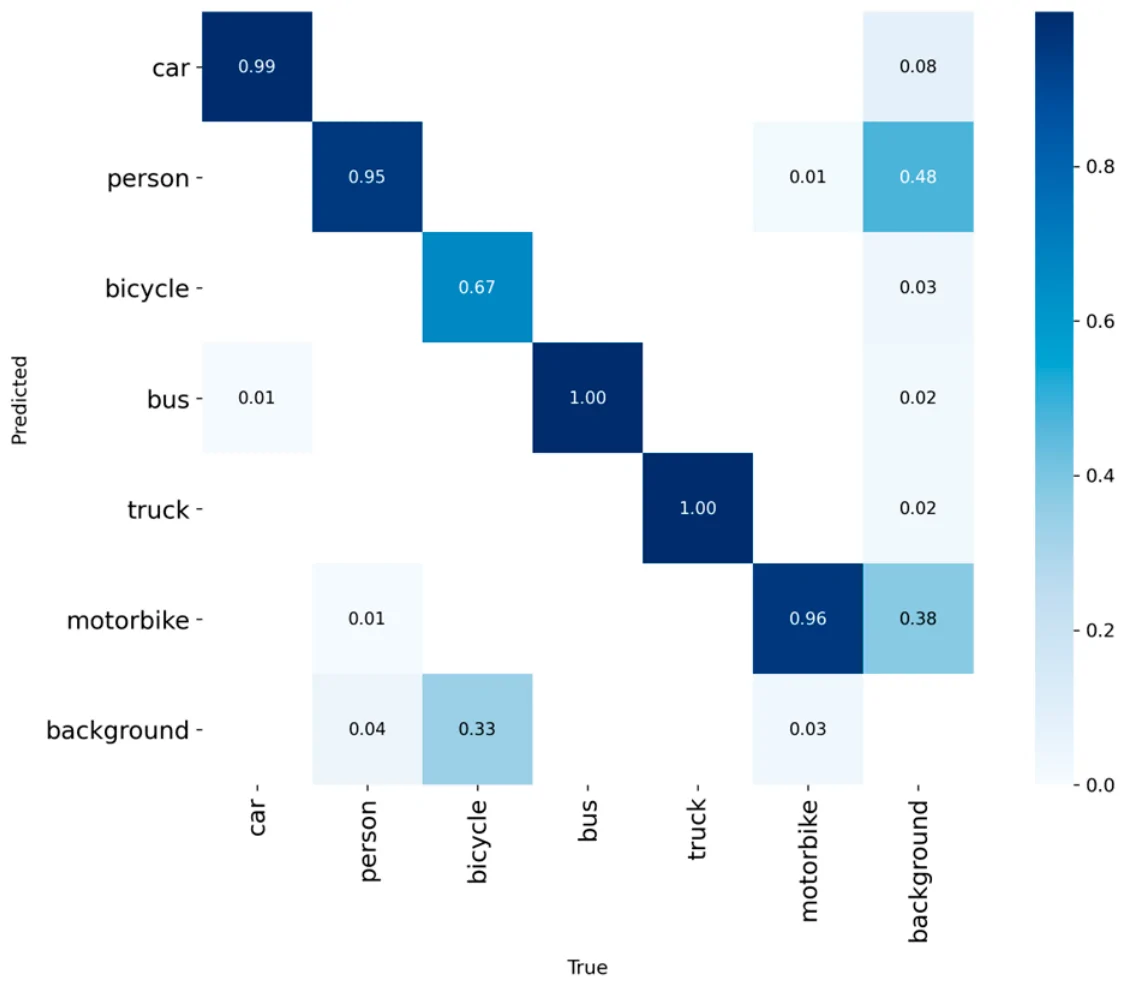
Normalized confusion matrix on UAV-MCT dataset.

The normalized confusion matrix of Aero-YOLO26 on UAV-MCT is depicted in Figure 8. The diagonal values indicate high recognition rates for person, motorbike, car, truck, and bus, while bicycle exhibits a lower recall than the other categories.

**Figure 9:**
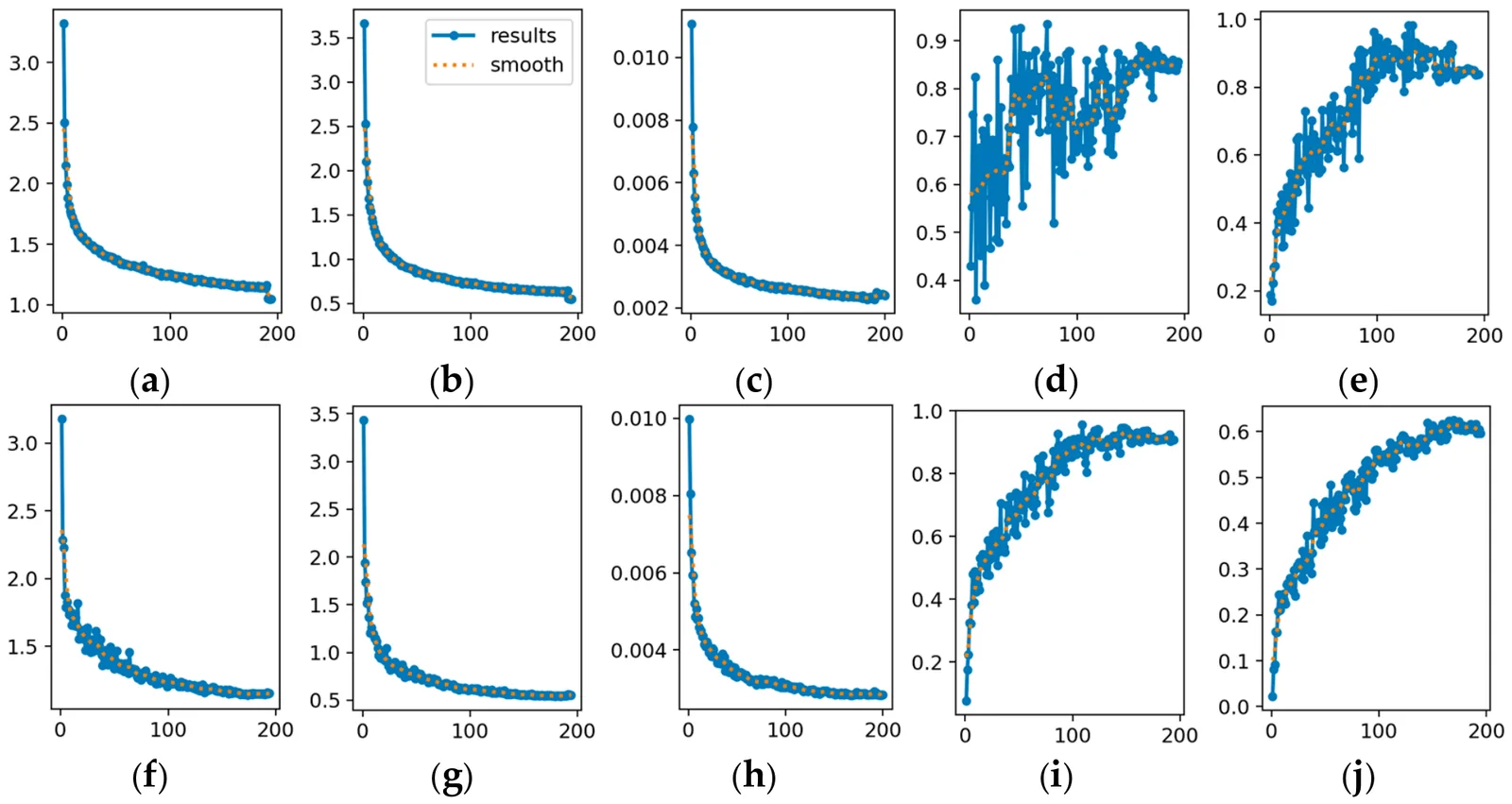
Training result graph of Aero-YOLO26 on UAV-MCT dataset: (**a**) train/box_loss; (**b**) train/cls_loss; (**c**) train/dfl_loss; (**d**) metrics/precision(B); (**e**) metrics/recall(B); (**f**) val/box_loss; (**g**) val/cls_loss; (**h**) val/dfl_loss; (**i**) metrics/mAP50(B); (**j**) metrics/mAP50-95(B).

Figure 9 presents the training and validation curves. The box and classification losses decrease during training, and the validation curves follow similar trends.

**Figure 10:**
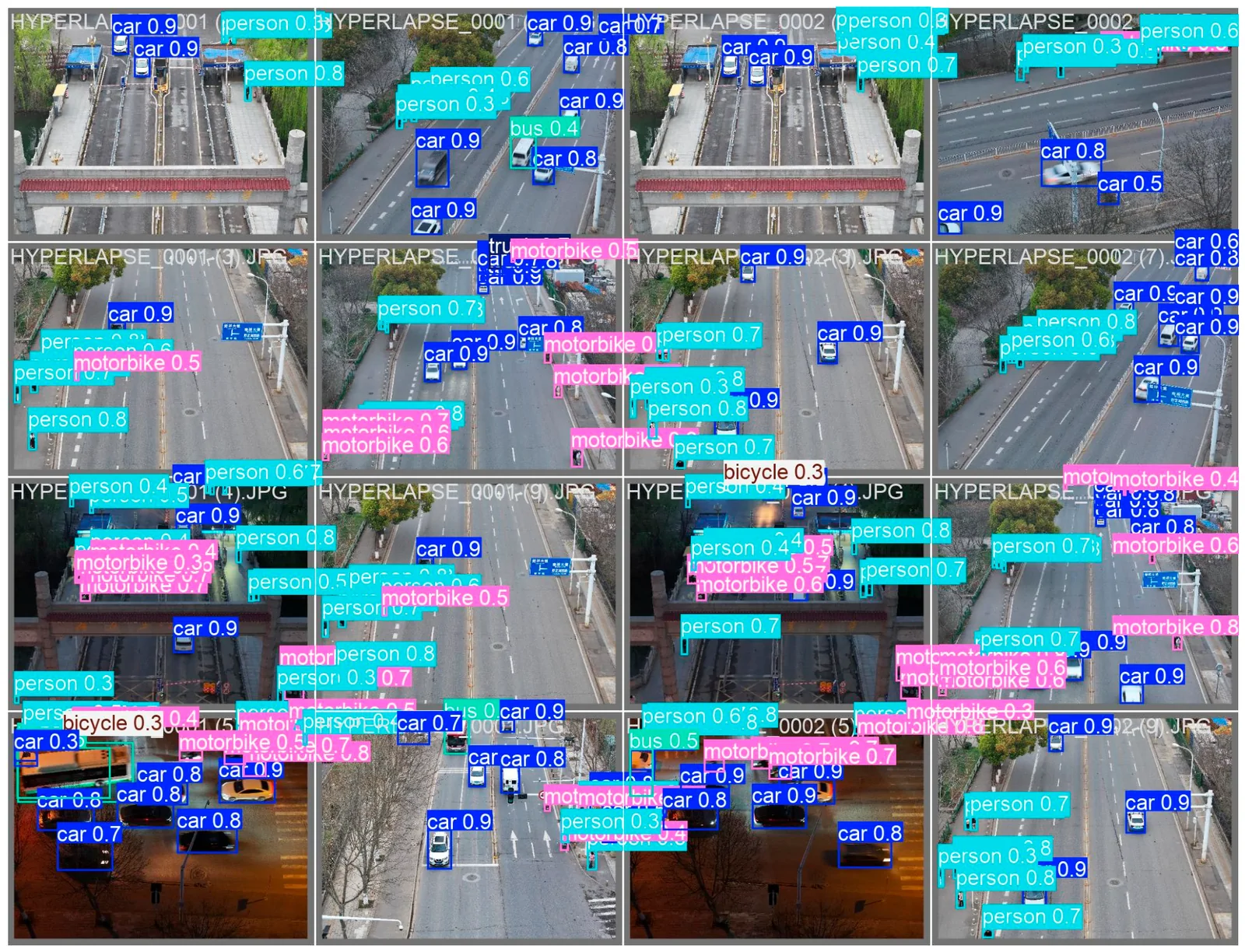
Example image of target detection results of Aero-YOLO26 on UAV-MCT dataset.

The qualitative detection examples in Figure 10 illustrate the predicted bounding boxes and categories in representative UAV-MCT scenes.

**Figure 11:**
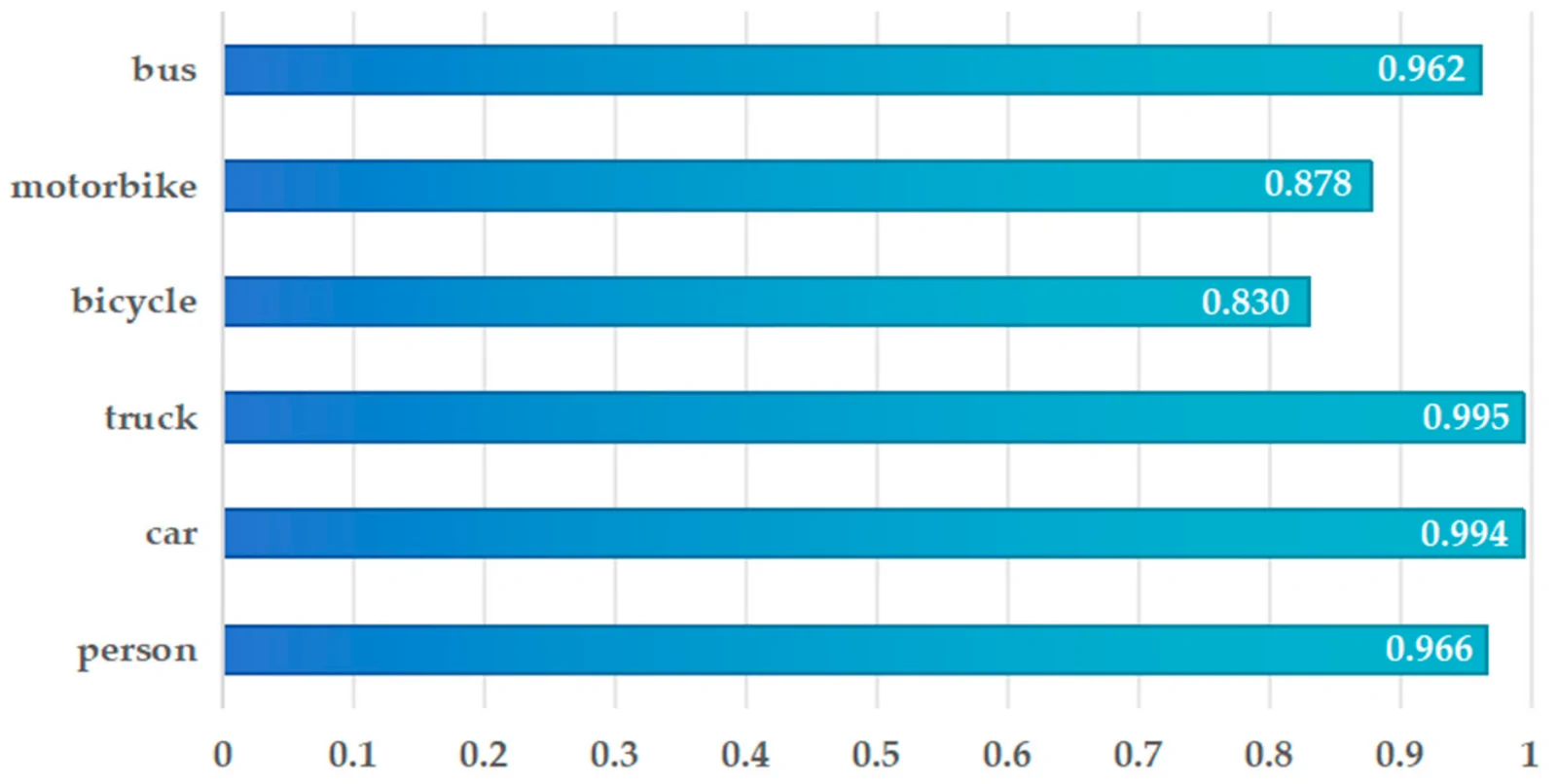
Per-class average precision on UAV-MCT dataset.

The AP performance comparison across categories is shown in Figure 11.

### 3.6. Ablation Study

For the purpose of evaluating and confirming the practical effectiveness of the proposed SlimSPPF and C2f_GhostECA modules on the object detection task based on UAV aerial photography, a step-by-step ablation study is conducted on the UAV-MCT dataset. To isolate the contributions of individual components, a series of architectural modifications are progressively introduced as follows: (1) Variant_A: all C2 modules are replaced by C2f_EnhancedECA while retaining the standard SPPF; (2) Variant_B: all C2 modules are replaced by C2f_Ghost while retaining the standard SPPF; (3) Variant_C: all C2 modules are replaced by C2f_GhostECA while retaining the standard SPPF; (4) Variant_D: only SPPF is replaced by SlimSPPF, with C2 left unchanged; (5) Variant_E: C2f_EnhancedECA is combined with SlimSPPF; (6) Variant_F: C2f_Ghost is combined with SlimSPPF; (7) Aero-YOLO26: the final model that integrates C2f_GhostECA with SlimSPPF. The ablation study was conducted using a separate configuration with a batch size of 4 and 100 training epochs, and the ablation experiment outcomes are illustrated in Table 8.

Compared with the baseline, Variant_A increases precision from 0.7719 to 0.9172, whereas Variant_B increases recall from 0.6743 to 0.9073. Variant_D improves mAP@50 from 0.7187 to 0.7826. The complete Aero-YOLO26 model achieves the highest mAP@50, mAP@50:95, and F1 score among the evaluated variants, while reducing the parameter count from 2.55 M to 1.99 M and GFLOPs from 27.8 to 22.5.

## 4. Discussion

### 4.1. Discussion of the Results of VisDrone2019 Dataset

In terms of the lightweight characteristics of the model, Aero-YOLO26 achieves extreme compression of parameter count by virtue of the C2f_GhostECA lightweight feature extraction module, the SlimSPPF lightweight multi-scale pooling module, and the reconstruction of the backbone network and PAN-FPN feature fusion network. The nano variant Aero-YOLO26n requires only 1.99 M parameters, making it the smallest model among all evaluated detectors. Compared with other nano-scale baselines, the parameter count of Aero-YOLO26n represents 63.0% of YOLOv8n, 93.4% of YOLOv9t, 76.0% of YOLOv11n, and 77.4% of YOLO26n. The small variant Aero-YOLO26s contains 9.16 M parameters, which is substantially lighter than YOLOv8s, YOLOv11s, and YOLO26s, corresponding to 82.0%, 96.8%, and 91.5% of their sizes, respectively. When compared with specialized UAV-oriented lightweight detectors, Aero-YOLO26n is 34.8% smaller than Drone-YOLOn, 48.5% smaller than BAF-Net, and merely one-fourth the size of YOLO-RACE. Despite integrating stronger feature representation, Aero-YOLO26s maintains a parameter count that is still comparable to the smaller Drone-YOLOt and far below YOLO-RACE. When compared with medium-scale models such as YOLOv8m and YOLO26m, both Aero-YOLO26 variants exhibit an order of magnitude reduction in parameter count. Thus, the computational and storage overhead is greatly reduced while retaining strong multi-scale receptive fields and feature extraction capabilities.

In terms of core detection accuracy, Aero-YOLO26 demonstrates a significant breakthrough on the basis of an extremely lightweight design. The nano variant Aero-YOLO26n achieves the best performance among the evaluated nano models, with a precision of 0.500, recall of 0.395, mAP@50 of 0.382, and F1 score of 0.441. Horizontally compared with nano baselines, it surpasses YOLOv8n by 10.86% in precision, 12.2% in recall, 7.9% in mAP@50, and 11.9% in F1 score. Compared with YOLOv9t, the absolute mAP@50 improvement of Aero-YOLO26n reaches 3.8 pp with an almost identical parameter count. Against specialized UAV-oriented lightweight detectors, Aero-YOLO26n outperforms Drone-YOLOn by 2.1 pp in mAP@50 and achieves an F1 score 3.8 pp higher, while Aero-YOLO26n demonstrates improvements over BAF-Net by 4.2 pp in mAP@50 and 4.0 pp in F1 score. Aero-YOLO26n also surpasses YOLO-RACE by a substantial 6.6 pp in mAP@50 with only 24.9% of the parameters, underscoring its superior accuracy–efficiency trade-off. Aero-YOLO26s achieves the highest precision, recall, mAP@50, mAP50-95, and F1 score among all small models. Horizontally, compared with YOLOv8s, Aero-YOLO26s delivers a 4.1 pp absolute gain; compared with YOLOv9s, YOLOv11s, and YOLO26s, the improvements are 2.9 pp, 4.6 pp, and 3.1 pp, respectively. Notably, Aero-YOLO26s also surpasses the dedicated Drone-YOLOt by 3.4 pp in mAP@50 and by 1.8 pp in F1 score, while using a comparable parameter budget. In a vertical comparison across scales, Aero-YOLO26s outperforms the medium YOLOv8m with only 35.4% of the parameters, and closely approaches YOLOv9m, YOLOv11m, and YOLO26m, trailing the strongest medium model by merely 2.7 pp while saving 58.2% of the parameters. Even when compared with the two-stage anchor-based detectors, Aero-YOLO26n already substantially outperforms Fast R-CNN and Mask R-CNN with less than 4.5% of their parameters, and Aero-YOLO26s delivers an even larger absolute mAP@50 improvement of over 12.7 pp against these traditional architectures. These gains are attributable to the C2f_GhostECA module, whose GhostBottleneck_ECA and EECA attention amplify multi-scale feature responses critical for small aerial objects, and to the SlimSPPF module, which maintains full multi-scale receptive field coverage and removes redundant intermediate feature memory overhead via pre-channel compression.

The stark performance gap between the two-stage anchor-based models (Fast R-CNN, Mask R-CNN) and the Aero-YOLO26 series can be fundamentally attributed to the divergence between anchor-based and anchor-free detection paradigms. Fast R-CNN and Mask R-CNN rely on densely pre-defined anchor boxes of various scales and aspect ratios to generate region proposals, which are then refined through a second-stage regression and classification. In UAV imagery, where objects exhibit extreme scale variation, heavy occlusion, and a predominance of small targets, such hand-crafted anchors struggle to provide adequate coverage and lead to severe foreground–background imbalance. Consequently, these methods incur massive computational overhead yet yield very low mAP@50. In contrast, Aero-YOLO26 adopts an anchor-free detection strategy, which discards the predefined anchor boxes and instead directly predicts object centers or key points along with their bounding box offsets. Thus, the design removes the sensitive hyperparameter tuning of anchors, simplifies the detection head, and naturally accommodates objects of arbitrary shape and scale aligned with the attributes of aerial targets. Coupled with the lightweight C2f_GhostECA and SlimSPPF modules, the anchor-free Aero-YOLO26n achieves a 26.9% higher mAP@50 than Fast R-CNN and a 21.7% higher mAP@50 than Mask R-CNN, while reducing the parameter count by more than 95%, and Aero-YOLO26s attains an mAP@50 12.7 pp higher than the best two-stage competitor. In all, the comparison substantiates that the anchor-free paradigm, when enhanced by attention-driven lightweight feature extraction, representing a more principled and efficient solution for UAV-based object detection than conventional anchor-based two-stage frameworks.

In terms of deployment efficiency, Aero-YOLO26n achieves the fastest ONNX throughput and the lowest latency among all evaluated detectors, surpassing the heavily optimized YOLOv8n and YOLOv11n. Aero-YOLO26s runs PyTorch inference at 62.93 FPS, comparable to the medium YOLOv8m, and delivers 103.8 ONNX FPS with 9.63 ms latency. The ONNX speed is 66% faster than YOLOv8m, 36% faster than YOLOv11m, and 37% faster than YOLO26m, while achieving substantially higher accuracy than the lightweight YOLOv8s. The resulting accuracy clearly demonstrates that Aero-YOLO26s provides an optimal trade-off; it retains real-time inference capability and offers medium-scale accuracy at a small-scale latency budget, a highly desirable property for airborne perception pipelines.

With respect to per-class performance, Aero-YOLO26n attains the highest AP on six of the nine VisDrone2019 categories. The gains are particularly pronounced on pedestrian and van; compared with the strongest competing nano model, the AP of pedestrian improves by 4.8 pp over YOLO26n, and the AP of van improves by 4.4 pp over YOLOv8n, underscoring the effectiveness of the EECA module in suppressing background clutter and highlighting small-scale objects. In contrast, Aero-YOLO26s attains the highest AP on five of the nine VisDrone2019 categories. The gains are particularly substantial on pedestrian and van, underscoring the effectiveness of the EECA attention in suppressing background clutter and highlighting small-scale objects. For both Aero-YOLO26n and Aero-YOLO26s, a modest decrease is observed on a few extremely tiny categories such as bicycle and awning tricycle, which aligns with the typical trade-off of lightweight architectures where the extreme parameter compression slightly attenuates the representation of severely scale-limited instances. Crucially, the dominant aerial categories that drive overall detection performance, such as car and pedestrian, exhibit clear and significant improvements.

Although the VisDrone2019 dataset provides a standardized basis for model comparison, its results alone cannot fully characterize the performance of real-world aerial photography scenarios. Therefore, the results of the UAV-MCT dataset are expected to be discussed as follows.

### 4.2. Discussion on the Results of UAV-MCT Dataset

To complement the benchmark comparison on the VisDrone2019 dataset, Aero-YOLO26 completes iterative training on the UAV-MCT dataset, and its performance, convergence characteristics, and scene adaptability are stable and excellent. For the small-scale group, the AP values for person, motorcycle, and bicycle are 0.966, 0.878, and 0.830, respectively, yielding a group-wise mean AP of 0.891. Although the non-motorized categories exhibit slightly lower figures due to their smaller physical dimensions and greater vulnerability to occlusion in cluttered aerial views, they still maintain high detection reliability, effectively overcoming the inherent difficulties of weak features and limited pixels. The medium-sized car attains an AP of 0.994, while the large object group, comprising truck and bus, averages 0.979, with individual values of 0.995 and 0.962. The relatively high mean AP of the small-object group suggests that the model retains useful sensitivity to small targets under the evaluated UAV-MCT conditions. This balanced performance across scales directly validates the SlimSPPF module, which successfully preserves a comprehensive multi-scale receptive field essential for handling the extreme scale variations inherent to UAV-captured scenes, achieving around a 30% reduction in peak pooling-branch memory access cost. Meanwhile, the recalibrated depth and width scaling coefficients in the reconstructed backbone and PAN FPN feature fusion network deliberately prioritize sensitivity to small instances, ensuring that fine-grained features are not diluted by larger contextual regions.

As shown in Figure 8, the recall diagonal entries indicate that person attains 95%, motorcycle is 96%, and bicycle approaches 67%. While bicycle’s recall is lower due to its smaller footprint and higher occlusion susceptibility, it still achieves an AP of 0.830, indicating that the model maintains strong discriminative ability despite moderate misses. Notably, person and motorcycle achieve recall rates on par with or exceeding those of medium and large categories, confirming that the model successfully retrieves the vast majority of small instances, a direct consequence of the EECA attention that suppresses background distractions while amplifying target-specific features. The confusion matrix (see Figure 8) also shows non-negligible off-diagonal responses for person and motorbike. These errors may be associated with their visual similarity, viewpoint variation, occlusion, and the imbalanced category distribution of the UAV-MCT dataset. Inter-class confusion among small categories is minimal, and bicycles exhibit virtually no cross-category errors, demonstrating that the model has learned highly discriminative features for slender and easily occluded targets. In contrast, buses and trucks exhibit near-perfect diagonal entries in the confusion matrix, consistent with their high AP values of 0.962 and 0.995, confirming that Aero-YOLO26 allocates discriminative capacity effectively across all scales, although bicycles remain more difficult than the other categories.

In terms of core detection indicators, the final precision of the proposed Aero-YOLO26 model reaches 85.61%, the recall rate is 83.78%, and the mAP50 and mAP50-95 reach 90.82% and 59.60%, respectively. The bounding box localization loss and classification loss exhibit a smooth, continuous descending trend throughout the training procedure. Both the training and corresponding validation losses converge synchronously, with no obvious oscillations or signs of overfitting. Notably, since the distribution focal loss module is removed in Aero-YOLO26, the train/val DFL remains consistently at zero across the entire training process, confirming the module’s deactivation. All small categories maintain high precision well above 0.9 even at elevated recall levels, with the curves for motorcycle and bicycle nearly covering the entire coordinate space, indicating an ideal trade-off between retrieval completeness and false positive suppression. In this regard, the behavior stems from the EECA attention, while the lightweight GhostConv structure prevents over-parameterization that could otherwise lead to overfitting. The mAP50-95 metric, which reflects localization accuracy under more stringent IoU criteria, reaches 59.60% with no significant scale-dependent degradation, confirming that bounding-box regression accuracy is well preserved for small instances. Thus, the proposed Aero-YOLO26 model completes efficient parameter optimization and feature learning during training and exhibits stable generalization ability.

Regarding model lightweightness and computational efficiency, the Aero-YOLO26 model maintains a low computational load in both training and inference stages. It enters a stable convergence state after 100 epochs of training iterations, and realizes a remarkable enhancement in detection precision avoiding a substantial increase in parameters or computation, aligning with the engineering application characteristics of UAV real-time detection.

Addressing the core challenges of dense small targets, significant scale differences, complex backgrounds, and easy target occlusion in UAV aerial photography scenarios, Aero-YOLO26 shows good scene adaptability. The high recall rate effectively reduces the missed detection rate in dense traffic flow and person flow scenarios, and the performance of the mAP50-95 indicator reflects precision detection ability on small objects under different IoU thresholds. The attention mechanism and receptive field optimization reinforce discriminative feature extraction for small objects, adapting to the scale diversity of the target and complex background clutter at different flight altitudes and shooting angles, maintaining stable detection performance under variable conditions.

In terms of the recognition accuracy performance of each category, the detection accuracy of Aero-YOLO26 for various targets in the UAV-MCT dataset presents the characteristics of excellent layering and overall balance. The AP values for trucks and cars reach 0.995 and 0.994, approaching ideal values, and the AP value of buses is 0.962. The three types of large and medium-sized vehicles all achieve high-precision detection, which verifies the feature extraction and positioning ability of the model for vehicle targets from diverse scales with poses from the UAV perspective. The AP value of the person target is 0.966, which effectively overcomes the detection difficulty of small size and weak features of people in an aerial photography perspective. The AP values of motorcycle and bicycle non-motor vehicle targets are 0.878 and 0.830, respectively. Although the accuracy is slightly lower than that of motor vehicles and people due to the smaller scale and vulnerability to occlusion, they still maintain high detection reliability in complex UAV aerial photography scenarios, with no significant shortcomings in the overall category accuracy.

In all, the results on the VisDrone2019 dataset and UAV-MCT dataset demonstrate the behavior of the complete Aero-YOLO26 model, but they do not isolate the contribution of each architectural component. Therefore, the ablation results are analyzed next to establish a more direct connection between module design and detection performance.

### 4.3. Discussion on the Results of Ablation Experiment

As revealed by the experimental data, substituting all C2 modules with C2f_EnhancedECA (Variant_A) yields a notable precision gain of 18.8% and increases mAP@50 and mAP50-95 by 12.1% and 15.7%, respectively, whereas recall slightly decreases by 2.4%. This indicates that the ECA mechanism significantly strengthens the discriminability for high-confidence positive samples at a minor cost in coverage. Meanwhile, the parameter counts drop from 2.55 M to 2.23 M and GFLOPs are reduced by 11.2%, confirming that the EECA design achieves lightweight feature representation while enhancing accuracy.

In contrast, replacing all C2 modules with C2f_Ghost (Variant_B) drives recall to 0.9073, a 34.6% improvement over the baseline of YOLO26, with mAP@50 reaching 0.8942 and mAP50-95 climbing to 0.6108. However, precision falls to 0.7674, which is lower than the baseline. This demonstrates that the Ghost module, through cheap linear transformations, generates redundant features that markedly improve multi-scale object coverage but somewhat weaken feature discriminability. The Variant_B becomes substantially more lightweight, with parameters and GFLOPs decreasing by 22.0% and 16.9%, respectively. The integration of Ghost and ECA in Variant_C restores precision to 0.9157 while achieving a recall of 0.7449, which outperforms Variant_B in precision and improves recall by 13.2% over Variant_A. With an mAP@50 of 0.8222 and an F1 score of 0.8215, all attained at a cost of only 1.99 M parameters and 23.1 GFLOPs, the variant achieves a balanced trade-off between feature accuracy and completeness, confirming that the GhostECA module effectively combines the precision-enhancing attention mechanism with the coverage-improving Ghost features, and enabling attention-enhanced lightweight feature extraction.

When SlimSPPF is introduced alone (Variant_D), precision rises to 0.8868, and mAP@50 improves by 8.9%, while recall shows no meaningful change and the overall network GFLOPs barely change. Although the standalone SlimSPPF module reduces peak memory access by about 30%, the global GFLOPs metric changes slightly because the SPPF module only accounts for a tiny fraction of the full network’s computational budget. This reveals that SlimSPPF refines the localization of salient objects through finer-grained multi-scale pooling, but its independent performance gain is mainly reflected in high-confidence positive samples and cannot substantially lift the recall of tiny targets alone.

Combining SlimSPPF with C2f_EnhancedECA (Variant_E) pushes recall to 0.9220, the highest among all variants, and mAP@50 to 0.8604. However, precision drops sharply to 0.6699, resulting in a conspicuous precision–recall trade-off and an F1 score of only 0.7760. This phenomenon suggests that SlimSPPF amplifies the ECA response in weak-response regions, causing the Variant_E to generate more positive predictions and thereby drastically boosting recall while suppressing precision.

The combination of C2f_Ghost and SlimSPPF (Variant_F) yields a moderate mAP@50 of 0.8694, which is slightly lower than that of Variant_B, while mAP50-95 declines from 0.6108 to 0.5869 and F1 also decreases. This indicates that superimposing the redundant Ghost features and the multi-scale context from SlimSPPF does not bring consistent gains. Instead, it introduces a certain degree of interference in localization accuracy.

Finally, the proposed Aero-YOLO26 deeply integrates C2f_GhostECA with SlimSPPF, provides a favorable accuracy–efficiency balance, and attains a precision of 0.8523, a notable recall of 0.8874, and the mAP@50 of 0.8968, representing a 24.8% improvement over the baseline of YOLO26. The mAP50-95 increases to 0.6234 (a 30.1% gain), and the F1 score reaches 0.8695, with all metrics standing at or near the optimum. Remarkably, these superior results are achieved with only 1.99 M parameters and 22.5 GFLOPs, corresponding to reductions of approximately 22.0% in parameter count and 19.1% in GFLOPs relative to the baseline. The final model retains the same parameter count as the Ghost-only variant while further reducing GFLOPs from 23.1 to 22.5. In all, these results verify that GhostECA offers complementarity between lightweight feature extraction and attention enhancement, and SlimSPPF can synergistically collaborate with GhostECA to achieve an excellent balance between precision and recall, substantially elevating the comprehensive detection capability.

### 4.4. Summary

Taken together, the three experiments provide complementary evidence for the advantage of the proposed Aero-YOLO26. The VisDrone2019 comparison evaluates the proposed model against representative two-stage detectors, mainstream YOLO-series models, and UAV-oriented lightweight detectors under a standardized benchmark. The UAV-MCT experiment further examines the performance of Aero-YOLO26 on an independently collected UAV image dataset containing diverse real-world urban scenes. Then, the ablation study provides a more direct explanation of how the proposed modules contribute to the observed detection behavior.

The results indicate that C2f_GhostECA and SlimSPPF address different aspects of lightweight aerial object detection. Ghost-based operations reduce redundant feature-generation cost and help preserve feature coverage under a limited parameter budget, EECA recalibrates channel responses and improves the selectivity of target-related features in complex backgrounds, and SlimSPPF retains multi-scale pooling-based contextual aggregation while reducing intermediate feature redundancy through channel pre-compression. The ablation results show that some variants favor precision, whereas others favor recall. As reflected by its highest mAP@50, mAP@50:95, and F1 score in the ablation experiment, the final Aero-YOLO26 configuration achieves the most balanced overall performance.

Moreover, the cross-dataset results are consistent with the intended design objective. On the VisDrone2019 dataset, Aero-YOLO26n improves the detection of several major aerial categories, including car, pedestrian, motor, and van, while maintaining a compact model size. On the UAV-MCT dataset, the Aero-YOLO26n model achieves strong performance for vehicle and person categories under independently collected urban UAV scenes. These findings suggest that the proposed Aero-YOLO26n architecture can preserve useful target representations under background clutter, scale variation, and partial occlusion. However, the weaker performance observed for bicycle and some other small or sparsely represented categories also shows that the proposed Aero-YOLO26n model does not completely eliminate the challenges associated with limited pixel coverage, occlusion, class imbalance, and inter-class visual similarity.

From an efficiency perspective, Aero-YOLO26n and Aero-YOLO26s provide different operating points; the nano variant prioritizes parameter efficiency and inference throughput, whereas the small variant provides higher detection accuracy at a greater computational cost. Together with the UAV-MCT evaluation, these results demonstrate strong potential for practical deployment in edge-side real-time object detection on UAV onboard platforms.

Nevertheless, the current experiments should not be interpreted as complete onboard deployment validation. The reported FPS and latency values are measured using a laptop GPU and ONNX Runtime, rather than an embedded UAV computing platform. Further evaluation is required to measure end-to-end latency, memory consumption, power usage, thermal behavior, communication overhead, and long-duration operational stability during flight.

## 5. Conclusions and Future Work

Aerial image target detection is confronted with key challenges including feature annihilation of small targets, large-scale variations, complex background interference, and limited onboard edge computing power. The mainstream YOLO26 model lacks targeted lightweight and small-target optimization for aerial photography scenarios, and it is challenging to reconcile model volume and detection accuracy. To address these issues, this study puts forward an Aero-YOLO26 lightweight aerial small-target detection algorithm and completes the full-link optimization based on the YOLO26 architecture. By designing the C2f_GhostECA lightweight feature extraction module, it integrates GhostConv and EECA attention to reduce parameters and improve efficiency.

On the VisDrone2019 dataset, the proposed Aero-YOLO26 series consists of two compact variants. The nano variant Aero-YOLO26n requires only 1.99 M parameters, making it the smallest model among all evaluated detectors, and achieves a precision of 50.0%, a recall of 39.5%, and an mAP@50 of 38.2%, comprehensively outperforming every other nano-scale detector as well as dedicated aerial detection methods such as BAF-Net. The small variant Aero-YOLO26s, with 9.16 M parameters, attains a precision of 55.1%, a recall of 42.7%, and an mAP@50 of 44.1%, ranking first across all five primary metrics among small-scale models. Notably, it surpasses the medium-scale YOLOv8m (43.8% mAP@50, 25.90 M parameters) while using only 35.4% of the parameters. On the UAV-MCT dataset, the proposed Aero-YOLO26 model has a precision of 85.61%, mAP@50 of 90.82%, and the AP values for car and pedestrian reach 99.4% and 96.6%, respectively.

Consequently, the proposed model achieves substantial gains in small-object detection from aerial imagery, along with an exceptionally lean parameter profile. With the advantages of lightweight deployment and high-precision detection capability, it has promising application prospects in two typical scenarios: smart city management inspection and autonomous driving airborne perception. For the smart city management inspection scenario, the model’s ultra-small parameter count with low computational load can perfectly adapt to the computing power limitations of UAV onboard edge devices.

Although Aero-YOLO26 has achieved improvements in lightweight and core accuracy, there are still some shortcomings. First, the precision of extremely small non-motorized targets such as bicycles, tricycles, and awning tricycles is low, and there is an insufficient balance of category detection. Second, the proposed model is only verified under conventional illumination and viewing angle conditions, and the robustness to extreme aerial photography scenarios such as low light, glare, motion blur, rain, and fog has not been fully verified.

Subsequent investigations will focus on optimizing the feature learning ability of extremely small and densely occluded targets to compensate for the performance gap in non-motorized small-target detection. At the same time, anti-interference feature enhancement modules and extreme scene data augmentation will be introduced to enhance the robustness in adverse environments. Moreover, the proposed model will be deployed to the real smart city aerial inspection scenario to verify the detection performance and practical engineering value in real scenarios.

## Figures and Tables

**Table 1:** Hardware and software configurations for experiments.

Device	Configuration
System	Windows 11
GPU	NVIDIA GeForce RTX 4060 Laptop GPU
CPU	13th Gen Intel (R) Core (TM) i9-13900HX
Python version	3.11.15
IDE	PyCharm 2025.3.3
Framework	PyTorch 2.6.0 + cu124

*Note:* The NVIDIA GeForce RTX 4060 Laptop GPU is manufactured by NVIDIA Corporation, Santa Clara, United States and the 13th Gen Intel(R) Core (TM) i9-13900HX is manufactured by Intel Corporation, Santa Clara, United States.

**Table 2:** The formulation of metrics.

Metric	Formulation
Precision (P)	$P=\frac{\mathrm{TP}}{\left(\mathrm{TP}+\mathrm{FP}\right)}$
Recall (R)	$R=\frac{\mathrm{TP}}{\left(\mathrm{TP}+\mathrm{FN}\right)}$
Average Precision (AP)	$\mathrm{AP}=\int_{0}^{1}p\left(r\right)\mathrm{dr}$ [28,32]
Mean Average Precision (mAP)	$\mathrm{mAP}=\frac{1}{k}\sum_{i=1}^{k}AP_{i}$
*mAP*_50_	*mAP*_50_ = *mAP^IoU^*^=0.5^
*mAP*_50−95_	$\mathrm{mA}P_{50-95}=\int_{0.5}^{0.95}\mathrm{mAPd}\left(\mathrm{IoU}\right)$
F1 Score	$F_{1}=2\cdot \frac{P\cdot R}{P+R}$

**Table 3:** Training parameter settings.

Methods	Setup
Batch size	8
Epochs	300
Learning rate	0.01
Momentum	0.937
Weight decay	0.0005
IoU	0.7
Box loss weight	7.5
Class loss weight	0.5
DFL weight	1.5

**Table 4:** Specific performance metrics of mainstream models on VisDrone2019 dataset.

Methods	P	R	mAP@50	mAP50-95	Params (M)	F1
Fast R-CNN	0.336	0.354	0.301	0.130	59.1	0.345
Mask R-CNN	0.432	0.316	0.314	0.171	44.4	0.365
YOLOv8n	0.451	0.352	0.354	0.205	3.16	0.394
YOLOv8s	0.510	0.390	0.400	0.237	11.17	0.442
YOLOv8m	0.550	0.424	0.438	0.262	25.90	0.479
YOLOv9t	0.455	0.340	0.344	0.199	2.13	0.389
YOLOv9s	0.519	0.400	0.412	0.245	7.32	0.451
YOLOv9m	0.558	0.431	0.446	0.268	20.22	0.486
YOLOv11n	0.442	0.336	0.339	0.196	2.62	0.381
YOLOv11s	0.496	0.396	0.395	0.233	9.46	0.440
YOLOv11m	0.567	0.439	0.452	0.272	20.11	0.495
YOLO26n	0.435	0.343	0.343	0.196	2.57	0.384
YOLO26s	0.539	0.385	0.410	0.245	10.01	0.449
YOLO26m	0.593	0.444	0.468	0.286	21.90	0.508
YOLO-RACE	0.419	0.321	0.316	0.180	8.0	0.364
Drone-YOLOn	0.465	0.356	0.361	0.203	3.05	0.403
Drone-YOLOt	0.521	0.416	0.407	0.221	5.35	0.463
BAF-Net	0.472	0.349	0.340	0.199	3.86	0.401
Aero-YOLO26n	0.500	0.395	0.382	0.219	1.99	0.441
Aero-YOLO26s	0.551	0.427	0.441	0.251	9.16	0.481

**Table 5:** Deployment efficiency metrics of mainstream models on VisDrone2019 dataset.

Methods	GFLOPs	FPS	Latency (ms)	ONNX FPS	ONNX Latency (ms)
YOLOv8n	8.9	141.7	7.06	258.5	3.87
YOLOv8s	28.8	171.2	5.84	145.6	6.87
YOLOv8m	79.3	63.4	15.77	62.5	15.99
YOLOv9t	8.5	50.5	19.79	111.0	9.01
YOLOv9s	27.6	49.2	20.34	91.5	10.93
YOLOv9m	77.9	54.2	18.45	55.2	18.13
YOLOv11n	6.6	130.1	7.69	214.1	4.67
YOLOv11s	21.7	134.3	7.44	149.2	6.70
YOLOv11m	68.5	74.4	13.43	76.4	13.10
YOLO26n	6.1	71.6	13.96	174.7	5.72
YOLO26s	22.8	71.3	14.02	128.9	7.76
YOLO26m	75.4	62.2	16.07	75.6	13.22
Aero-YOLO26n	22.5	144.4	6.94	264.3	3.78
Aero-YOLO26s	89.9	62.93	15.89	103.8	9.63

**Table 6:** Per-class AP of nano-scale models on VisDrone2019 dataset.

Category	YOLOv8n	YOLOv9t	YOLOv11n	YOLO26n	Aero-YOLO26n
car	0.776	0.769	0.772	0.762	0.807
pedestrian	0.384	0.369	0.378	0.392	0.440
motor	0.400	0.390	0.391	0.398	0.450
van	0.399	0.392	0.388	0.374	0.443
truck	0.317	0.292	0.288	0.314	0.319
bicycle	0.243	0.091	0.093	0.089	0.126
bus	0.488	0.486	0.442	0.465	0.485
tricycle	0.243	0.228	0.210	0.216	0.255
awning tricycle	0.134	0.134	0.121	0.131	0.132

**Table 7:** Per-class AP of small-scale models on VisDrone2019 dataset.

Category	YOLOv8s	YOLOv9s	YOLOv11s	YOLO26s	Aero-YOLO26s
car	0.810	0.806	0.811	0.808	0.831
pedestrian	0.457	0.455	0.454	0.483	0.500
motor	0.467	0.474	0.464	0.477	0.496
van	0.432	0.447	0.441	0.438	0.502
truck	0.361	0.385	0.341	0.380	0.344
bicycle	0.144	0.150	0.140	0.155	0.124
bus	0.566	0.598	0.549	0.591	0.555
tricycle	0.279	0.291	0.267	0.292	0.297
awning tricycle	0.147	0.164	0.146	0.156	0.177

**Table 8:** Ablation experiment of Aero-YOLO26 on UAV-MCT dataset.

Methods	P	R	mAP@50	mAP50-95	F1	Params (M)	GFLOPs
Baseline	0.7719	0.6743	0.7187	0.4792	0.7198	2.55	27.8
Variant_A	0.9172	0.6579	0.8058	0.5546	0.7662	2.23	24.7
Variant_B	0.7674	0.9073	0.8942	0.6108	0.8315	1.99	23.1
Variant_C	0.9157	0.7449	0.8222	0.5731	0.8215	1.99	23.1
Variant_D	0.8868	0.6620	0.7826	0.4862	0.7580	2.55	27.2
Variant_E	0.6699	0.9220	0.8604	0.5691	0.7760	2.23	24.7
Variant_F	0.7458	0.8301	0.8694	0.5869	0.7857	1.99	22.5
Aero-YOLO26	0.8523	0.8874	0.8968	0.6234	0.8695	1.99	22.5

## Data Availability

Data will be made available on request.
